# Growth rates of marine prokaryotes are extremely diverse, even among closely related taxa

**DOI:** 10.1093/ismeco/ycae066

**Published:** 2024-05-02

**Authors:** Ona Deulofeu-Capo, Marta Sebastián, Adrià Auladell, Clara Cardelús, Isabel Ferrera, Olga Sánchez, Josep M Gasol

**Affiliations:** Departament de Biologia Marina i Oceanografia, Institut de Ciències del Mar, CSIC, Barcelona, Catalunya 08003, Spain; Departament de Biologia Marina i Oceanografia, Institut de Ciències del Mar, CSIC, Barcelona, Catalunya 08003, Spain; Institut de Biologia Evolutiva, CSIC-UPF, Barcelona 08003, Catalunya, Spain; Departament de Biologia Marina i Oceanografia, Institut de Ciències del Mar, CSIC, Barcelona, Catalunya 08003, Spain; Centro Oceanográfico de Málaga, Instituto Español de Oceanografía, IEO-CSIC, Puerto Pesquero s/n, Fuengirola 29640, Málaga, Spain; Departament de Genètica i de Microbiologia, Universitat Autònoma de Barcelona, Bellaterra, Catalunya 08193, Spain; Departament de Biologia Marina i Oceanografia, Institut de Ciències del Mar, CSIC, Barcelona, Catalunya 08003, Spain

**Keywords:** marine prokaryotes, growth rate, amplicon metabarcoding, manipulation experiment

## Abstract

Marine prokaryotes play crucial roles in ocean biogeochemical cycles, being their contribution strongly influenced by their growth rates. Hence, elucidating the variability and phylogenetic imprint of marine prokaryotes' growth rates are crucial for better determining the role of individual taxa in biogeochemical cycles. Here, we estimated prokaryotic growth rates at high phylogenetic resolution in manipulation experiments using water from the northwestern Mediterranean Sea. Experiments were run in the four seasons with different treatments that reduced growth limiting factors: predators, nutrient availability, viruses, and light. Single-amplicon sequence variants (ASVs)-based growth rates were calculated from changes in estimated absolute abundances using total prokaryotic abundance and the proportion of each individual ASV. The trends obtained for growth rates in the different experiments were consistent with other estimates based on total cell-counts, catalyzed reporter deposition fluorescence *in situ* hybridization subcommunity cell-counts or metagenomic-operational taxonomic units (OTUs). Our calculations unveil a broad range of growth rates (0.3–10 d^−1^) with significant variability even within closely related ASVs. Likewise, the impact of growth limiting factors changed over the year for individual ASVs. High numbers of responsive ASVs were shared between winter and spring seasons, as well as throughout the year in the treatments with reduced nutrient limitation and viral pressure. The most responsive ASVs were rare in the *in situ* communities, comprising a large pool of taxa with the potential to rapidly respond to environmental changes. Essentially, our results highlight the lack of phylogenetic coherence in the range of growth rates observed, and differential responses to the various limiting factors, even for closely related taxa.

## Introduction

Marine prokaryotic communities exert a large influence on ocean ecosystem functioning, driving biogeochemical cycles. They change over multiple timescales in response to both biological interactions and environmental change. Their activity, structure, and function are largely dependent on the growth rate of individual prokaryotes. Hence, elucidating the variability and phylogenetic imprint of marine prokaryotes' growth rates are crucial for understanding microbial community dynamics and the role of individual taxa in global biogeochemical cycles [1]. This knowledge is essential for accurately modeling the responses of ocean ecosystems to environmental changes [2] and for defining appropriate timescales to study marine prokaryotic communities.

The growth rates of microorganisms can be broadly categorized into gross and net growth rates. Net growth rates represent *in situ* or actual (i.e. effective) growth rates (under a given condition), while gross growth rates represent the growth rate in the absence of all limiting factors. Gross growth rates can be approached through manipulation experiments that reduce or remove the limiting factors, and net growth rates can be calculated from the changes in the unaltered natural sample. Growth is limited by different processes, and the factors that limit growth rates can be classified into intrinsic, which are specific to the microorganism, and extrinsic factors, which include biotic, such as predation and viral infection, as well as abiotic factors such as light, temperature, and the availability of nutrients. Thus, the breadth of prokaryotic growth rates can be estimated through manipulation experiments that reduce or suppress these extrinsic factors. Currently, not a single method is perfect to determine growth rates. Bulk approaches, based on leucine or thymidine incorporation, estimate community averaged growth rates, without considering the heterogeneity in metabolism of marine prokaryotes [3]. Some studies have calculated growth rates of specific populations using catalyzed reporter deposition fluorescence *in situ* hybridization (CARD-FISH) [4–11] or flow cytometry [12]. At a high taxonomic resolution and using metagenomic data, maximal potential growth rates can be estimated through the computation of codon usage bias [13, 14], and *in situ* rates through the so-called “peak to trough” method [15, 16]. These two methods have been compared in a separate study [17], which concluded that the peak to trough method cannot reliably predict actual growth rates for most marine bacterial populations. In addition, growth rates have also been estimated through amplicon read normalization using internal standards (ARNIS) in manipulation experiments, a method which has increased the resolution to the 16S RNA gene amplicon sequence variants (ASV) level [18]. That study assessed the role of predators and phosphorus limitation in controlling the growth of individual ASVs [18]. However, viruses, light or other resource limitations, as well as less controlled environmental changes associated to ecosystem seasonality, have also a strong impact on growth rates [4, 19]. This has not been explored at a high resolution level yet, and the relationship between growth rate and phylogeny is still unclear.

We conducted incubation experiments to determine the breadth of growth rates of marine prokaryotes at the ASV level in the absence, or reduction, of various limiting factors such as nutrient limitation, grazers and viruses, and light availability during the four astronomical seasons. The main goal of the study was to understand whether growth rate is a phylogenetically determined trait. Additionally, we outlined several secondary objectives: (i) to explore the diversity of prokaryotic growth rates in a marine site, and their variations throughout the year, (ii) to examine the consistency of growth rate distributions across different taxonomical ranks, (iii) to investigate if the factors affecting growth rates are consistent among phylogenetically related taxa, (iv) to determine whether different treatments select for the same or different ASVs over the year, and (v) to analyze the relationship between growth rates and *in situ* abundances of individual taxa.

## Materials and methods

Surface water was collected from the Blanes Bay Microbial Observatory (BBMO), a shallow (~20 m depth) costal station at ~one km offshore in the NW Mediterranean Sea (41°40’N, 2°48′E) during the four astronomical seasons of 2017, as detailed in [6]. Environmental data are summarized in Table 1. The description of the experimental setup can be found in the Supplementary information. Briefly, the set up included six different treatments: unmanipulated seawater incubated under natural light/dark (CL) or dark (CD) conditions, a predator reduced treatment (<1 μm) incubated under natural light/dark (PL) or dark (PD) conditions, a diluted treatment reducing resource limitation (DL) and a virus-reduced treatment (VL). As an inevitable consequence of confinement of microbial communities is the so-called “bottle effect” [20], the control treatment may not reflect *in situ* conditions, but do serve as a control to assess the effect of the different growth limiting factors on the growth rates of individual taxa. Physicochemical conditions during the experiments are available at Table S1. Heterotrophic prokaryotes, picophytoplankton and viruses were quantified by flow cytometry and heterotrophic nanoflagellates using epifluorescence microscopy, as described in [6].

**Table 1:** Environmental physicochemical and biological parameters of the *in situ* community at the different sampling dates of the different experiments.

**Date**	**Season**	**Temperature (°C)**	**Salinity**	**Secchi disk depth (m)**	**Surface PAR (μmol photons m^-2 s^-1)**	**Chlorophyll *a* (μg L^-1)**	**[PO43-] (μM)**	**[NH4+] (μM)**	**[NO2-] (μM)**	**[NO3-] (μM)**	**[SiO4-] (μM)**	**DOC (μM)**	**Prokarayotic abundance (cells mL ^-1)**	**Bacterial production (μg C L^-1 d^-1)**	**Leu-based prokaryotic specific growth rate (d^-1)**	**% HNA prokaryotic cells**	**Heterotrophic nanoflagellate abundaance (cells mL ^-1)**	***Synechococcus* abundance (cells mL^-1)**	**Picoeukaryote abundance (cells mL ^-1)**	**Viral abundance (viruses mL ^-1)**
20-02-2017	Winter	12.8	38.01	8	546	1.2	0.044	0.214	0.28	1.17	1.51	63.8	1.04E+06	2.57	0.033	61.6	1.24E+03	1.06E+04	1.61E+04	9.89E+06
25-04-2017	Spring	14.8	38.06	20	569	0.43	0.028	1.57	0.119	0.357	1.19	65.7	1.01E+06	3.03	0.047	48.0	1.65E+04	4.43E+04	6.44E+03	1.16E+06
04-07-2017	Summer	23.1	38.02	20	789	0.13	0.015	0.431	0.036	0.034	0.69	86.2	7.28E+05	4.62	0.139	46.6	1.49E+03	1.70E+04	1.27E+03	7.75E+06
06-11-2017	Fall	19.5	37.7	19	224	0.46	0.025	0.2	0.04	0.155	0.663	77.9	1.58E+06	1.34	0.032	26.9	1.03E+03	3.45E+04	2.38E+03	1.09E+07

### DNA extraction and sequencing

Microbial biomass was concentrated onto 0.2-μm polycarbonate filters after sieving through a 20-μm mesh using a peristaltic pump. Between 2–4 L of water were filtered for *in situ* and t_0_ samples, and 1–2 L from each replicate of all treatments along the course of the experiments (t_2_, t_3_, t_4_). DNA was extracted (following [21]), purified and concentrated using Amicon 100 columns (Millipore) and quantified in a NanoDrop-1000 spectrophotometer (Thermo Scientific). The DNA was stored at −80°C, and an aliquot of each sample was used for metabarcoding and metagenome sequencing. Primers 515F-Y (5′-GTG YCAG CMG CCG CGG TAA) and 926R (5′-CCG YCA ATT YMT TTR AGT TT) [22] were used to amplify the V4-V5 regions of the 16S rRNA gene. The metabarcoding dataset were sequenced at the Integrated Microbiome Resource (IMR, Halifax, Canada, https://imr.bio/), using a MiSeq sequencer (2 × 250 bp, Illumina), but 29 samples that failed were re-sequenced at RTL Genomics (Lubbock, TX, USA, http://rtlgenomics.com/). The *in situ* natural community samples were sequenced at AllGenetics (A Coruña, Spain, www.allgenetics.eu). All of them followed the same procedures. A total of 312 samples were sequenced of which 66 (t_0_ and t_4_ of all treatments in three experiments (all but fall)) were selected for metagenome sequencing using an Illumina NovaSeq 6000 machine (Centre Nacional d’Anàlisi Genòmica, CNAG) with paired-end fragments of 150 bp, which provided on average 115 million reads (min = 67 M, max = 238 M) each. The winter and summer experiments had two replicates for t_4_, whereas the spring experiment presented three replicates. We used illumina-utils [23] for quality filtering the short reads from the metagenomes with the *iu-quality-minoche* function (default parameters), which removes noisy reads [24]. Find detailed sampling hours for each experiment in Fig. S1.

### ASVs and mOTU generation

ASVs were obtained from metabarcoding data using Dada2 [25], and taxonomic assignation performed against the SILVA database release 138 (see SI for further details). Metagenomic data, with no PCR bias, were also analyzed. We used mOTUs2 v3 pipeline [26] to obtain species profiles for each metagenome. Briefly, the method maps all the reads to a reference database based in the genome taxonomy database using a set of single copy genes [27], it calculates the relative abundance of each taxonomic group and determines the unassigned fraction.

### Single-ASV-based growth rate calculation

Based on the temporal changes in microbial community composition we were able to calculate single-ASV-based growth rates as follows: the relative abundances (%) of individual ASVs were multiplied by total prokaryotic abundances (obtained through flow cytometry) in each sample and divided by a hundred, to obtain pseudoabundances (cells/ml) for each individual ASV. Then, we calculated a lineal regression between time and ln-transformed pseudoabundances using values of the three replicates (see example in Fig. S2). By assuming that exponential growth was happening between t_0_ and t_3_ or t_4_, the regression slope indicates growth rate (time^−1^). As abundances may get saturated with time and the slope including t_4_ may be lower than that up to t_3_, we compared t_0_-t_2_-t_3_-t_4_ and t_0_-t_2_-t_3_ slopes in all regressions and the highest and more significant (*P*< .05) for each ASV at each condition (treatment and season) was chosen to determine maximal growth rates. Overall, we were able to calculate 3601 growth rates for 1287 individual ASV, with an estimated mean growth rate value of 2.55 ± 1.53 d^−1^. A total of 15 145 linear regressions (from 1612 ASVs) were discarded because they were neither significant nor positive. The reason why we discarded negative growth rates is related to the compositional nature of our data, which makes it impossible to differentiate ASVs that were dying during the experiment from slow growers that get displaced by the fast-growing community. Indeed, typical slow growers like SAR11 accounted for a large fraction of the calculated negative growth rates (Table S2), and most orders displaying negative growth rates did not belong to the growing community (Fig. S3). This supports our view that negative growth rates were in many cases an artifact derived from the compositional data.

To test for the potential bias in abundances due to differences in 16S rRNA gene copy numbers, we normalized the reads by their gene copy number using the rrnDB *Estimate* from the rrnDB Classsifier tool [28], before calculating growth rates and compared it with the unnormalized ones.

### Group abundance-based growth rate calculation

We additionally compared our results with the group abundance-based values presented in Sanchez *et al*., 2020 [6]. 4′-6-diamidino-2-phenylidole (DAPI) counts time-course measurements were used to estimate bulk growth rates, and CARD-FISH to explore the growth rates of different bacterioplankton phylogenetic groups. To compare the results of the CARD-FISH identified groups and the ASV-based growth rates, we first checked the specificity of the used probes with Testprobe 3.0 (https://www.arb-silva.de/search/testprobe/). Then, we pooled single-ASV growth rates of those phylogenetic groups that matched the results from Testprobe 3.0 into each of the CARD-FISH probes used. Next, we calculated the mean growth rate for each condition of each phylogenetic group. With these values, we computed the correlation between CARD-FISH abundance-based growth rates and pooled ASV-based growth rates.

### mOTUs-based growth rate calculation

Since we only had metagenomes from t_0_ and t_4_ (with replicates), but not time-course samples, we calculated the fold change of mOTUs abundance and transformed them into growth rates. First, we calculated mean relative abundances between replicates and multiplied them by total prokaryotic abundance using the flow cytometry data. Then we calculated the fold change dividing t_4_ by t_0_ data and converted it to growth rate:


$$Growth\ rate\left({d}^{-1}\right)=\frac{\mathit{\ln}\ \left( fold\ change\right)}{t_4-{t}_0}\ast 24$$

### Patterns of growth rate distribution at different taxonomic ranks

To explore the variability of the obtained prokaryotic growth rates, we summarized their patterns by plotting their distributions (i.e. density plots of the number of prokaryotic growth rates at each growth rate value). These distributions were plotted for different taxonomic ranks to examine the phylogenetic coherence of growth rates across different taxonomic levels. We analyzed those phyla for which we had obtained at least two values of growth rate over all the experiments.

### Growth rate response to limiting factors

To investigate the factors controlling growth rates and determine if they are consistent among phylogenetically closely related taxa, we compared ASV-based growth rates in the different treatments and seasons. We summarized four effects of different growth controlling factors as following: the light effect was estimated by calculating the difference between growth rates in the light and dark treatments, respectively (CL-CD/ PL-PD), top-down controls were divided in two different groups: the one imposed by viruses, represented by the difference in growth rates between the virus reduced and diluted treatments (VL-DL), and the one imposed by grazers, comparing predator reduced with control treatments, respectively (PL-CL/PD-CD). Finally, the bottom-up control, related to nutrient availability, was estimated through the comparison of the diluted and predator-reduced treatments (DL-PL). Additionally, we performed the same analysis at the family and order level, to test if there was a consistent response at higher taxonomic levels.

### Analysis of responsive ASVs in the different treatments and seasons

To investigate if the same ASVs were responding to the same treatments over the course of the year, we quantified the number of exclusive and shared ASVs that responded to each condition. To be considered a responsive taxon, a growth rate >1 d^−1^ was established as a threshold. Exclusive ASVs were defined as those that responded to only one condition, while shared ASVs between two conditions, were those that responded to both conditions. We calculated these parameters in absolute and relative numbers. The relative number of exclusive ASVs per condition was calculated by dividing the number of uniquely responding ASVs to a condition by the total number of ASVs that responded to that condition. Accordingly, the relative number of shared ASVs was determined by dividing the number of shared ASVs between two conditions by the number of maximum possible common responding ASVs (i.e. the lowest number of responsive ASVs from the two conditions considered).

### Relationship between *in situ* abundance and growth

To evaluate if the most-responsive taxa were abundant or rare in the initial natural communities we first had to relate the *in situ* community ASVs sequences to the ones used to calculate the growth rates in these experiments. In the case that a responsive ASV was not detected in the natural community we assumed that it was present but undetected due to methodological constrains. We were able to relate 595 ASV, which accounted for 46.2% of the growing community. We categorized as rare-most-responsive taxa those ASVs that represented <1% in the natural community and displayed a growth rate > 2 d^−1^ in the experiments, and very rare most-responsive-taxa the ones that represented <0.1% in the natural community and displayed a growth rate >2 d^−1^. Moreover, we considered taxa as successful rare-most-responsive taxa when they ended up representing >1% of the relative abundance in the community at the end of the experiment. We determined the threshold of 2 d^−1^ growth rate as fast growth because it is twice the average rate reported in the literature from 26 seawater cultures [1].

### General data analyses

All analyses were run in R software v. 4.0.5 [29] and Rstudio software v. 2022.12.0 + 353 [30] using these main libraries libraries: phyloseq v. 1.38.0 [31] and tidyverse v. 1.3.1 [32]. For statistical analyses we used: FSA v. 0.9.3 [33] rstatix v. 0.7.0 [34] and Hmisc package [35]. Other packages used are detailed in the SI. Statistical comparison of growth rates at different conditions were analyzed as follows; first, normality was tested with the Shapiro test. Then, if normality was confirmed, we used a parametric Tukey HSD test, whereas in case of no normality, we used a non-parametric Kruskal test and Dunn test. The Pearson or the Spearman methods were used for correlations in the case of normality, and lack of normality, respectively.

## Results

Of the 2899 ASV present at the initial times of the incubation experiments, we were able to calculate growth rates for 1287 ASVs, resulting in 3601 growth rate values corresponding to different treatments and seasons (Table S3). The percentage of the growing community (i.e. number of ASVs displaying growth divided by initial (t_0_) richness) was 44%, with a mean growth rate value of 2.56 ± 1.53 d^−1^ and a median of 2.23 d^−1^.

### Large variability in the growth rates values of individual marine prokaryotic ASVs

**Figure 1:**
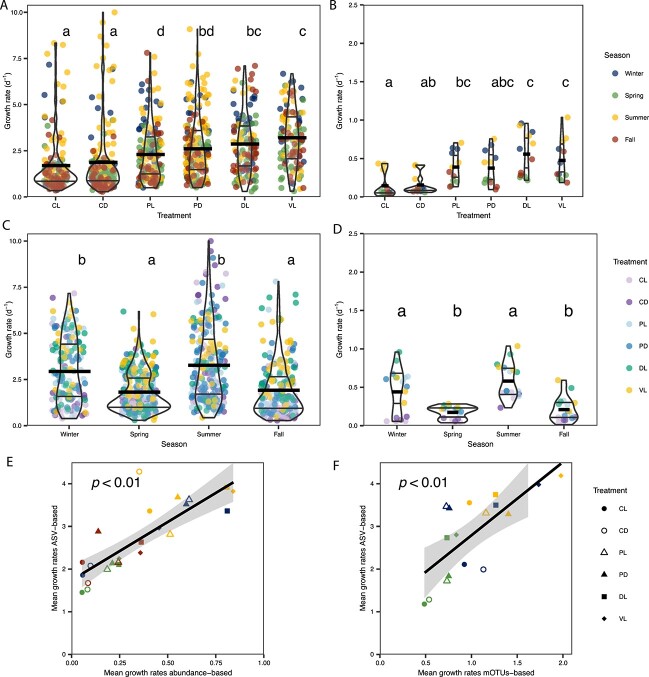
Growth rate changes across different treatments and seasons and comparison with other methods. Treatment abbreviations meaning: The first letter corresponds to the treatment (C: Control, P: Predator reduced, D: Diluted and V: Virus reduced) and the second letter corresponds to the treatment light regime (L: Day-night cycles, D: Darkness). Violin plots of A) single-ASV-based growth rates in the different treatments, B) cell-abundance-based estimated growth rates in the different treatments, C) single-ASV-based growth rates in the different seasons, D) cell-abundance-based estimated growth rates in the different seasons. In A and B, the colors correspond to the different seasons, whereas in C and D, colors correspond to the different treatments. The Y axis represents the growth rate (d^−1^) in all panels, but note that the scale in a and C is four times higher than in B and D. Violin plots indicate density distribution, wide lines show mean values, and thin lines show quartiles. Letters indicate statistically different groups. The mean prokaryotic abundance-based growth rates derive from [6]. E) Correlation between mean prokaryotic-abundance-based growth rates (X axis) and mean growth rates per treatment and season of single-ASV-based growth rates (Y axis). We used the Spearman method since normality was not confirmed with the Shapiro test (*P* < .01). Note that the Y axis is four times the X axis. F) Correlation between mean growth rates based on mOTUs and ASV-based growth rates per treatment and season. The Pearson method was used since normality was confirmed with the Shapiro test (*P* < .01). Only those ASV/mOTUs that represented at some point >1% of the community were included in these analyses. In panel F, only those ASV-based growth rates that were calculated using t_4_ are used.

Single-ASV-based growth rates presented a huge variability within treatments and seasons, with values ranging from ~0.3 to almost 10 d^−1^. The top 20 single-ASV-based growth rates values are summarized in Table S4. Average growth rate increased as we reduced cumulative controlling factors, such as predators (PD: 2.61 ± 1.61 d^−1^ and PL: 2.3 ± 1.53 d^−1^), nutrient limitation (DL: 2.86 ± 1.58 d^−1^) and viruses (VR: 3.2 ± 1.53 d^−1^) (Fig. 1A). The highest variabilities were measured in the control treatments (CD and CL, coefficient of variation (CV): 97% and 92%, respectively) (Fig. 1A). Overall, bulk community growth rates derived from total cell counts showed a similar trend (but lower absolute values) than single-ASV-based growth rates (Fig. 1B), except for the VL treatment which showed slightly lower mean growth rates than the DL treatment (Fig. 1B). Regarding the different seasons, single-ASV-based growth rates were highest in summer (3.26 ± 2.07 d^−1^), with high variability (CV: 63%), followed by winter (2.93 ± 1.75 d^−1^, CV: 60%), and lower and similar mean values in spring (1.81 ± 1.07 d^−1^, CV: 59%) and fall, which had the highest coefficient of variation (1.91 ± 1.45 d^−1^, CV: 76%) (Fig. 1C). Trends were also consistent with the growth rates obtained from cell-counts (Fig. 1D).

Given that 16S rRNA tag sequencing values might be biased because of variability in rRNA operon copy numbers in different organisms, we compared the estimated single-ASVs growth rates values with those corrected by 16S rRNA copy number (see methods). Corrected and uncorrected values had a similar range and the correlation between the two estimates was significant (Spearman value = 0.9, N = 3601, *P* < .05, Fig. S4). Yet, to avoid the noise introduced by a correction that is at best approximate [36], subsequent analyses were performed with unnormalized abundances.

### Comparison of single-ASV-based growth rates with abundance-based and mOTU-based growth rates

Single-ASV-based average growth rates per condition correlated significantly with the growth rates based on total cell-abundance counts (Spearman value = 0.85, N = 24, *P* < .05), but comparatively were four times higher (Fig. 1E). Similarly, single-ASV based average growth rates per condition correlated significantly with mOTU-based average growth rates (Pearson value = 0.75, N = 17, *P* < .05, Fig. 1F), but were ~two times higher. The correlation between averaged (per treatment and season) single-ASV-based and CARD-FISH-based growth rates was also significant (*N* = 24, *P* < .05) for Eubacteria, Rhodobacterales (probe ROS537), Cytophaga-Flavobacteria (probe CF319), and Gammaproteobacteria (probe GAM42a), but not significant (*P* > .05) for Alteromonadales (ALT1413) and the gammaproteobacterial NOR5 clade (NOR5–730) (Table S5, Fig. S5). However, the high growth rates observed for the *Alteromonadaceae* family are consistent with the high group-specific values observed in [6].

### Changes in growth rate distribution as we increase the taxonomic resolution

**Figure 2:**
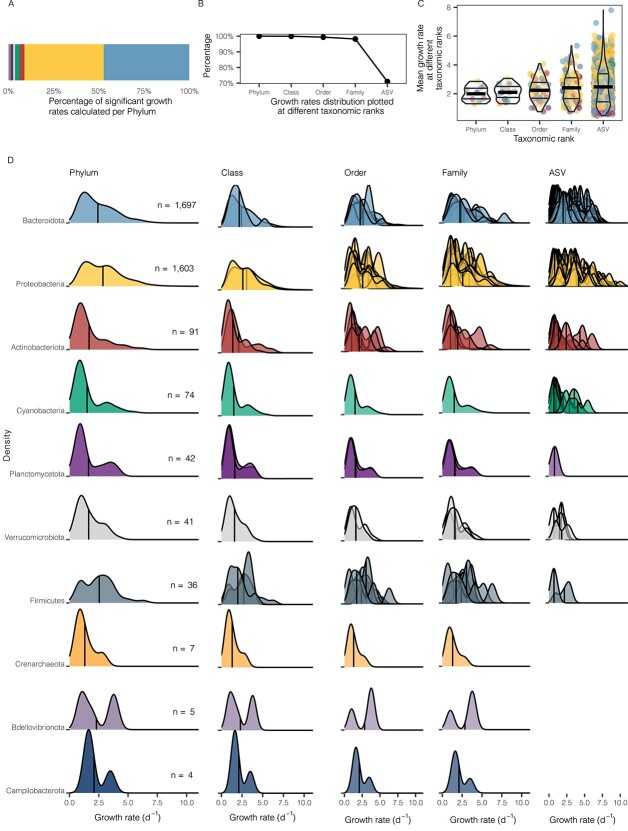
Growth rate distribution patterns at different taxonomic ranks for all treatments and seasons. A) Taxonomic assignment at the phylum level of the ASVs that displayed significant growth rates. B) Percentage of growth rate values plotted for the different taxonomic ranks. C) Violin plots presenting growth rates at different taxonomic ranks for all treatments and seasons, dots are colored by phylum, thin lines represent quartiles and the wide lines the mean. D) Growth rate distribution at different taxonomic ranks: The X axis presents growth rate (d^−1^), the Y axis presents the density distribution pattern for each taxonomic rank that presented at least two significant growth rates in the whole study, and the vertical line represents the mean. Each column indicates the taxonomic rank used for grouping the distributions plotted, and each row represents a phylum. The n value in panel D informs of the number of growth rates represented in each phyla. Density plots in which the minimal observations per group (two) at this taxonomic rank is not reached are not presented in the ASV level column.

The great majority of single-ASV-based growth rates that were calculated belonged to Bacteroidota ASVs (*n* = 1697) and Proteobacteria ASVs (*n* = 1603) (Fig. 2A). Note that we only plotted distributions when we had at least two observations (per taxonomic rank). Thus, when we decreased the taxonomic rank (from phylum to ASV), we were able to plot less distributions (Fig. 2B), with the lowest amount at the ASV taxonomic level, where only 72% of the growth rates could be considered. The average growth rates observed at the different taxonomic ranks were quite constant, but the variability increased at high taxonomic resolution (Fig. 2C). Accordingly, the patterns of distribution of all growth rates calculated during the incubation experiments showed that as we increased the taxonomic resolution, different distributions of growth rates arose (Fig. 2D). In the first column, the patterns plotted at the phyla level were different for each phylum: bimodal-like (Campilobacterota and Bdellovibrionota), skewed right (Bacteroidota, Actinobacteriota, Cyanobacteria, Planctomycetota, and Verrucomicrobiota), or more normal-like (Proteobacteria and Firmicutes). At the class level some different distributions emerged within some of the phyla, as in Firmicutes, which presented a peak around 4 d^−1^ for one class, but a smoother distribution for other classes. More multimodal distributions appeared at the order and family level (Fig. 2D) in all phyla with more than three orders or families plotted. At the highest resolution, i.e. at the ASV level, growth rate distribution patterns became wider**,** particularly for Proteobacteria and Bacteroidota, which displayed an enormous range of ASV-based growth rates (0.29–9.99 d^−1^). We also explored how the growth-rate distributions at the family level varied in the different treatments for those phyla with high number of observations (Fig. S6). Overall, the global trend of higher growth rates as we decrease the growth limiting factors (Fig. 1A) was also observed at the family level (Fig. S6).

**Figure 3:**
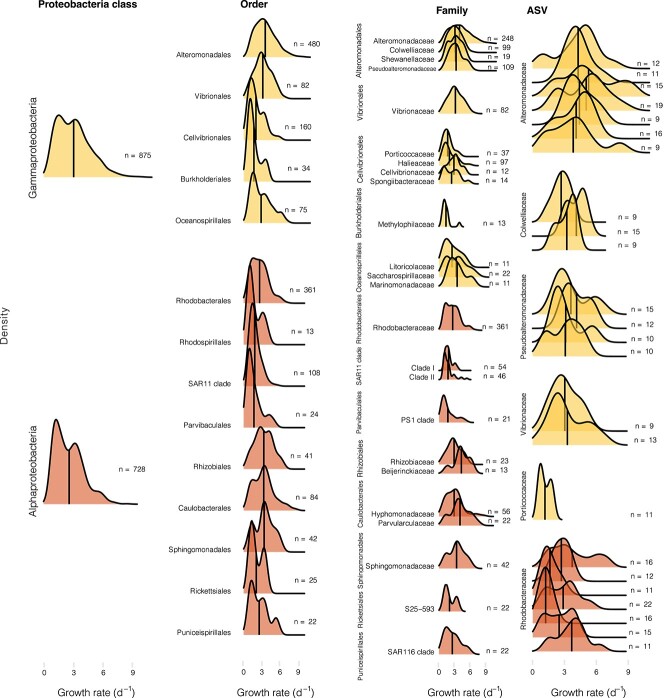
Growth rate distribution patterns within the Proteobacteria phylum. Each column represents a different taxonomic level; from left to right: Class, order, family and ASV, the X axis shows growth rates (d^−1^), and the Y axis presents the density distribution pattern for each taxonomic group. The dataset was filtered with the following parameters: n values >10 for classes, orders and families, and >8 for ASVs. These filters were used to simplify the plot visualization.

As Proteobacteria and Bacteroidota represented a large proportion of growing ASVs, we further explored them in detail (Fig. 3 and Fig. S7, respectively). Among the Proteobacteria, both Gammaproteobacteria, and Alphaproteobacteria presented a slightly bimodal distribution in growth-rates. Within the Gammaproteobacteria, at the order level, Alteromonadales and Vibrionales had a normal-like distribution pattern, with mean values around 3 d^−1^, whereas the other orders presented growth rates skewed towards lower values (Fig. 3). Looking at the ASV level we could appreciate that some ASV presented multimodal distributions. Notable differences were found at the ASV level within the *Rhodobacteraceae* family, which showed a wide distribution in the first ASV, but normal-like and multimodal distributions in other ASVs. In the SAR11 Clade, each family presented a characteristic distribution but a similar average, although Clade II showed some higher growth rates, of up to 5.36 d^−1^. Among the Bacteroidota phylum, the *Flavobacteriaceae* family had a notably broad growth rate range at the ASV level, from 0.3 to 7.5 d^−1^ (Fig. S7).

### Effect of growth limiting factors on taxonomically closely related taxa

**Figure 4:**
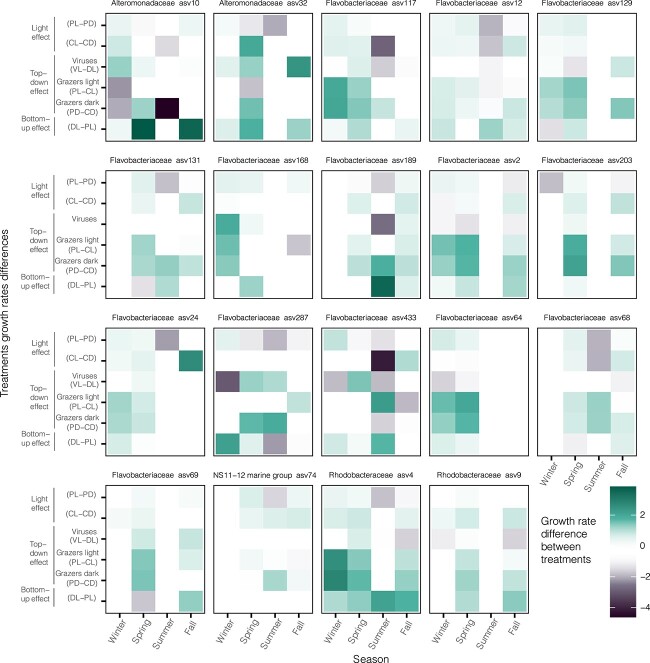
Summary of treatment effects on growth rate values of different ASVs. The X axis presents seasons, and the Y axis the difference between the mean growth rate from different treatments, indicated by varying color. Summary of values in SI (Table S6). Treatment abbreviations meaning: The first letter corresponds to the treatment (C: Control, P: Predator reduced, D: Diluted and V: Virus reduced) and the second letter corresponds to the treatment light regime (L: Day-night cycles, D: Darkness).

We investigated whether the factors controlling the growth rates are consistent among closely related taxa and if the same factors control the growth rates of individual ASV across seasons (see methods for details). Overall, the mean community response across all ASVs growth rates was close to 0 for all limiting growth factors, although the removal of light availability in summer had a highly negative impact on growth rates (Fig. S8), in agreement with the observations in Sanchez et al., 2020 [6] using cell counts data. This lack of community response reflected the substantial variability in growth rate responses to the various treatments among ASVs, families, and orders (Figs 4, S9, and S10; data summarized in Tables S6, S7 and S8, respectively, see SI for a detailed description of the trends). To provide a comprehensive overview, we present only the ASVs, families, and orders with a high number of comparable values between seasons and treatments. We observed that for all ASVs, families and orders there was a remarkable variation on the effects of each limiting factor depending on the season, especially for *Flavobacteriaceae* ASVs (Fig. 4). The general trend was that each ASV was affected differently by the different factors over the year, and no taxonomic coherence was observed for different ASVs belonging to the same family. For example, the two *Alteromonadaceae* ASVs were affected differently by the controlling factors (Fig. 4). In contrast, we identified some taxonomic coherence for ASVs belonging to the *Rhodobacteraceae* family: ASV4 was highly positively impacted by the removal of the top-down control almost all the year, similarly to its closely related ASV9 in spring and fall (Fig. 4).

### Similarities between responsive ASVs across treatments and seasons

**Figure 5:**
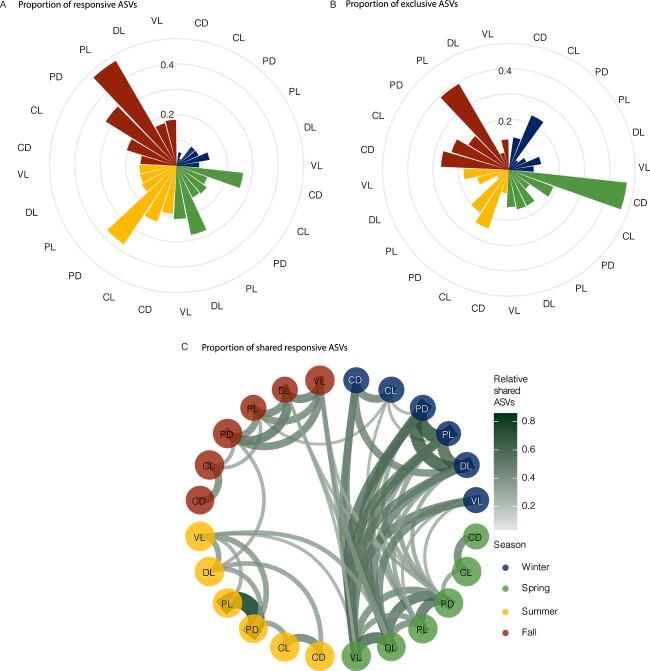
Analysis of responsive ASVs in the different treatments and seasons. We considered that an ASV was responding to a treatment if it presented a growth rate > 1 d^−1^. A) Relative number of responsive ASVs from the total number of ASVs present in each sample at t_0_. B) Exclusive ASVs growing in each season and treatment in relation to the total ASVs growing in that condition. C) Proportion of shared ASVs between treatments and seasons, line width and color represent the relative number of shared ASVs between these two samples. Only when the proportion of ASVs shared between conditions was higher than 30% connecting lines were plotted to simplify the visualization. Colors denote the different seasons. Treatment abbreviations meaning: The first letter corresponds to the treatment (C: Control, P: Predator reduced, D: Diluted and V: Virus reduced) and the second letter corresponds to the treatment light regime (L: Day-night cycles, D: Darkness).

To test the potential consistency of microbial responses to different treatments over the year, we quantified the number of exclusive (i.e. in one treatment and season only) and shared (i.e. in more than one treatment and season) responding ASVs (defined as having growth rates >1 d^−1^) for each condition (treatment and season). The proportion of responsive ASVs was lower in winter and highest in the predator reduced treatments in fall (Fig. 5A, see Fig. S11A for absolute number of responsive ASVs). The proportions of exclusive ASV (those growing in a single season and treatment) varied among treatments, but were generally <20%, with some exceptions: in spring in the CD treatment (~50% of the ASVs) and fall in the PL treatment (~40% of the ASVs, Fig. 5B and Fig. S11B). Shared responsive ASVs (number of common responsive ASVs in two treatments) were highest between winter and spring (Fig. 5C and Fig. S11C). Conversely, summer and fall exhibited the fewest shared responsive ASVs, with only a few shared responsive ASVs between spring DL and VL treatments and summer VL treatments. Control treatments generally shared a higher number of ASVs within the same season than across seasons. Same pattern was observed for the PR treatments, except for the winter season, where they shared a high number of ASVs with spring DL and VL treatments. In contrast, the DL and VL treatments shared many responsive ASVs across different seasons (Fig. 5C and Fig. S11C).

### Relationship between *in situ* abundance and growth

**Figure 6:**
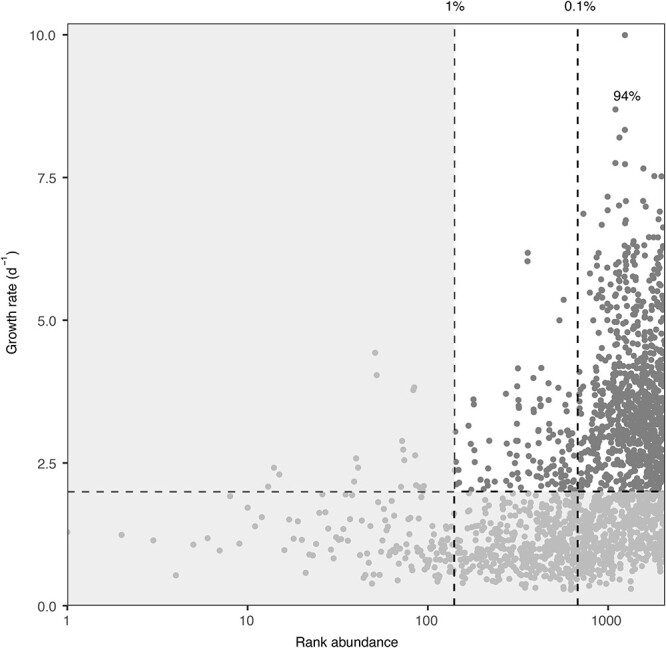
Relationship between relative abundance in the natural community and growth rates in the incubation experiments. The x-axis presents the ranked taxa based on their relative abundance in the *in situ* communities (where 1 is the most abundant one) and the y-axis the growth rate (d^−1^). Note that the x-axis is log-transformed to facilitate visualization. Dashed vertical lines indicate relative abundance at 1% and 0.1% of the natural community, and horizontal dashed lines a growth rate of 2 d^−1^. The number inside the plot indicates the percentage of very rare-most-responsive ASVs within the rare-most-responsive ASV.

We next explored whether the most responsive ASVs were initially rare or abundant in the *in situ* communities. We considered “most responsive ASVs” those ASVs that displayed growth rate values >2 d^−1^, doubling the previous threshold of responsive ASVs (1 d^−1^), which is the growth rate threshold for copiotrophic taxa in the marine environment [1]. We observed that the vast majority of these most responsive ASV in the experiments (320 ASVs) were initially rare (<1%). Moreover, most of them (301, ~94%) were very rare (<0.1% at initial communities) (Fig. 6). The same analysis was performed separately for seasons and treatments, with similar results (Fig. S12). These most responsive ASVs represented on average <10% of the ASVs in the *in situ* communities. Of the 320 most responsive ASVs, 44% ended up representing >1% of community sequences at the end of the experiments (Fig. S13).

## Discussion

Our study represents the first year-round and high-resolution assessment of the variability of growth rates in marine plankton prokaryotes. By analyzing amplicon metabarcoding time-course data from incubation experiments, we were able to calculate a wide range of growth rates and gain a broad perspective on the diversity of this trait in the marine environment. This methodology has proven to be a cost-effective and efficient method for calculating experimental growth rates at high-resolution, as previously demonstrated [18, 37]. Here, we calculated specific growth rates, using different treatments that cumulatively removed controlling factors (see [4] for a thorough discussion on the potential limitations of our manipulation approach for the different growth limiting-factors). Our metabarcoding approach is not exempt from biases, but those have been carefully considered (discussed in detail in the SI). One of the inevitable intrinsic biases of experimental manipulations is the so-called “bottle effect”, therefore the growth rates estimated in the controls may not reflect actual *in situ* growth rates. Yet, the experimental approach is still valid to explore the breadth of growth rates of different taxa which was the main goal of this study.

The observed growth rates reached values almost up to 10 d^−1^, which are four fold higher than rates estimated using changes in total cell counts in the same experiments [6]. However, total cell abundance-based growth rates include all the cells in the community, even those that are not growing, which often represent >40% of the community [38]. In contrast, by using single-ASV-based growth rates, we are only considering those microorganisms that are growing, which could explain the significant increase in the mean growth rates estimated. In addition, given the compositional nature of our metabarcoding data the growth of slow growers get masked by the fast growth of other members of the community, making it impossible to accurately calculate the growth rates of slow-growers. This would result in our growth rates values potentially skewed towards fast growers, which may explain why our reported maximal growth-rate values are almost two fold the values previously estimated using metabarcoding or metagenomes in manipulation experiments [17, 18]. However, our growth rate values are in the range of observations in the Blanes Bay Microbial Observatory during nutrient-addition experiments [39], or values reported for particle-associated communities [40], and lower than some estimations obtained with the codon usage technique [13]. When comparing the ranges of growth rates observed in our experiments with those in [18] at the genus level, no significant disparities were detected. Some genera exhibited higher ranges in ASV-based estimation, while others showed higher ranges in the ARNIS-based method (Table S9). The observed differences likely stem from variations in environmental conditions across the experiments, resulting in the enhanced growth of different taxa (Fig. S14).

The pooled growth rates of broad phylogenetic groups have been useful for understanding growth rate variability among groups and over time in marine prokaryotic communities [4, 9, 10]. However, when working with these integrated values, we are missing an important part of the diversity within this trait, as values may change widely even among closely phylogenetically related taxa (Fig. 2 and [18]). For instance, within the typical slow-growing SAR11 clade, some ASVs from Clade II displayed notable growth rates (up to 5.4 d^−1^, Fig. 3). Although these estimated growth rates seem too high for a typical oligotroph, SAR11 have been occasionally found to display high growth rates, characteristic of copiotrophs, in nature [10, 40].

**Figure 7:**
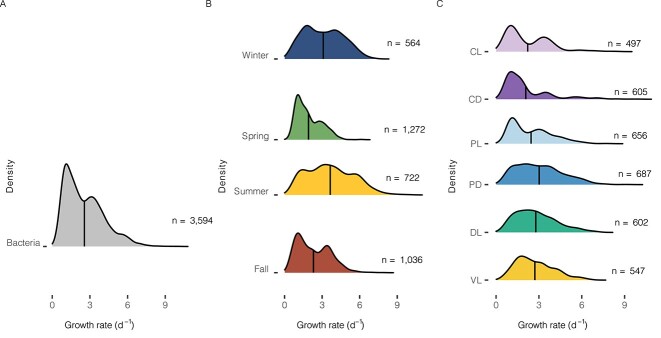
Growth rate distribution patterns at the domain level for all growth rates calculated during this study. Vertical black lines represent mean values, the X axis shows growth rates (d^−1^), and the Y axis presents the density distribution pattern for each condition. A) Bacterial growth rate distribution patterns. B) Bacterial growth rate distribution patterns grouped and colored by season. C) Bacterial growth rate distribution patterns colored by the different treatments explored in this study; CL: Control light, CD: Control dark, PL: Predator reduced light, PD: Predator reduced dark, DL: Diluted light and virus reduced light.

Growth rate distribution patterns have been related to prokaryote life strategies. In our case, bacterial growth rate distributions were mainly not bimodal (Fig. 7), contrary to the maximal potential growth rates estimated using the codon usage technique [13]. When working with predicted maximal growth rates, prokaryotes can present genomic imprints which lead to a natural division between oligotrophs and copiotrophs [13]. However, as expected, when measuring actual rates, this division would become diffuse, with most prokaryotic taxa falling in a range between the two. Indeed, our growth rate values presented a distribution near normal-like, in a continuum range, challenging the classification into one or two simple life-styles, as shown before [18, 41].

When analyzing the factors limiting the growth of different prokaryotes over the year, we observed that there was no taxonomic coherence, and seasonality appeared as a strong driver of growth rates. Some exceptions were the Rhodobacterales, as they were highly affected by top-down control grazers over most of the year, in agreement with previous observations [4, 19]. In contrast, although Altermonadales are also known to be grazed intensively [18], they displayed a strong seasonal pattern in growth rates, and no taxonomic consistency (Fig. 4). Growth of all *Flavobacteriaceae* ASVs showed changes in the limiting factors over the year, consistently with their observed seasonality [42]. Contrary to some reports [12], we observed significant growth in the virus-reduced treatment already within 36–48 h, indicating that viruses may have a rapid effect on prokaryotic growth (see for instance *Sphingomonadaceae* in Fig.S9)*.*

Winter and spring shared responsive ASVs among the different treatments (Fig. 5), possibly because both seasons are characterized by low nutrient limitation and cooler waters [6]. The virus-reduced and predator-reduced treatments had a notable overlap of responsive ASVs between seasons, implying year-round top-down pressure on the same ASVs (Fig. 5).

Our analyses also unveiled that most responsive taxa were recruited from the rare biosphere [43, 44], suggesting they awaited favorable environmental or ecosystemic conditions to thrive. However, roughly half of these ASVs became abundant (>1% of relative abundance) by the end of the experiments implying that only a fraction of the responsive taxa may eventually dominate the community forming a bloom. These taxa have been referred before as conditionally rare taxa [45] or rare reactive taxa [46]. While some ephemeral blooms have been captured in natural environments [47–51], further research is needed to understand the impact of these blooms on ecosystem dynamics and function. Furthermore, some of these rare ASVs could have been highly active *in situ*, and thus contribute to a large fraction of total production, consistently with previous observations [4, 12, 18].

To conclude, our study explored the breadth of growth rates of marine prokaryotes and whether there is taxonomic coherence in this metabolic trait. The significant correlation between the rates calculated using different approaches indicate that our results are sound independently of potential methodological biases. Yet, ASV-based rates are higher than those calculated by other methods, as it only considers those ASVs that are actively growing. No general phylogenetic coherence in the range of growth rates was observed, even for closely related taxonomic groups, nor in the responses to the various limiting factors. Our data unveil high dynamism within microbial communities, with the presence of some rare taxa with growth rates reaching up to 10 d^−1^ that may produce ephemeral blooms [49], and have a significant impact in global biochemical cycles [52]. Most temporal monitoring programs sample on a weekly, monthly or seasonal basis at best, missing the intrinsic short-term variability of prokaryotic communities. The fact that some ephemeral blooms of rare taxa have occasionally been captured during these monitoring programs (e.g. [47, 49]) reinforces the need of studying microbial communities at short-temporal scales to fully comprehend ocean’s microbial dynamics.

## Supplementary Material

Supplementary_material_Deulofeu-Capo_et_al_topublish_ycae066

Supplementary_tables_Deulofeu-Capo_et_al_ycae066

## Data Availability

Sequence data have been deposited in ENA under accession number PRJEB60085. The R-scripts used to calculate growth rates, generate figures and statistical analyses are available at: https://github.com/onadeulofeu/Single-ASV-based_prokaryotes_growth_rates.
