# Supplementary material for: Growth rates of marine prokaryotes are extremely diverse, even among closely related taxa
Source: ISME Commun. 2024 May 2;4(1):ycae066. doi: 10.1093/ismeco/ycae066 (PMC11126302; doi:10.1093/ismeco/ycae066)
Supplement: Supplementary_material_Deulofeu-Capo_et_al_topublish_ycae066 [file supplementary_material_deulofeu-capo_et_al_topublish_ycae066.pdf]

Supplementary Information

Growth rates of marine prokaryotes are extremely diverse, even among closely related taxa

Authors: Ona Deulofeu-Capo, Marta Sebastián, Adrià Auladell, Clara Cardelús, Isabel Ferrera, Olga Sánchez and Josep M Gasol

Index

Supplementary methods **2**

Methodological limitations of the approach chosen **3**

Supplementary results **4**

References **6**

Supplementary tables **9**

Supplementary figures **11**

Supplementary methods

1.- Experimental mesocosms set up

The manipulation experiments are described in detail in [1], and they started the next day after sampling. Each experiment had triplicates of six different treatments, which cumulatively reduced factors known to control prokaryotic growth. In that order, i) the treatment control (CT): incubation of unmanipulated seawater; ii) the predator reduced (PR): seawater prefiltered through 1-μm filter to remove most predators; iii) a diluted treatment (DL): in which seawater was diluted 1:4 with 0.2-μm filtered seawater to reduce both predation and competition for nutrient and organic carbon resources; and iv) a virus-reduced treatment (VL): whole seawater diluted 1:4 with seawater filtered through a 30-kDa VivaFlow (tangential flow filtration) cartridge to reduce predation, viruses and resource competition. The DL and VL treatments were exposed to natural light/dark cycles, but the CT and PR treatments had two sub-treatments: natural light/dark cycles (CL and PL), and complete darkness (CD and PD), respectively. The light treatments were limited to photosynthetically active radiation (PAR) by maintaining the bottle incubations under natural light conditions with the exclusion of UV radiation, using two layers of an Ultraphan URUV Farblos Filter and a net that reduced light intensity to roughly mimic the light conditions encountered at a depth of 3 m, calculated from the transparency measures at the sampling site. PAR was monitored continuously in the incubation water baths. The dark treatments were completely covered with two layers of black plastic bags to prevent light exposure. The experiments were performed in eight L Nalgene bottles per triplicate, which were incubated in 200 L baths with circulating seawater, to maintain temperatures close to *in situ* conditions, and during 36/48 h (until cell abundance reached the stationary phase), and sampled every 12 h approximately. For each treatment and experiment, a total of five samples were taken for nucleic acid extraction and cell counts: one right after seawater sample collection to characterize the *in situ* natural community; at t_0 of the incubation experiments; and approximately every 12 h (t_2, t_3, t_4). Additionally, one sample was taken after six h of incubation (t_1), but only for the estimation of cell abundance.

2.- Metabarcoding processing

For the metabarcoding analyses of the experiments, we generated three different runs of sequences (from the manipulation experiments: the ones sequenced at IMR and the ones that failed, and another one from the natural community) that needed to be processed separately. Initially, we used cutadapt [2] to trim primers. Then, the ASV table was obtained running dada2 1.18.0 version [3], using the pool method to increase sensitivity to sequences that may be present at very low frequencies in multiple samples, keeping a mean of 82.7%± 0.1 sequences per sample. Taxonomic assignation was performed with DECIPHER 2.16.1 version [4] at 60% confidence aligning against the SILVA database release 138. Four samples with <5,000 reads were discarded, keeping a total of 308 samples for further analyses. To assess the *in situ* abundance of ASVs growing in the manipulation experiments, we merged datasets from the manipulation experiments and the one from the natural community using the function cluster_fast from VSEARCH v.2.17.0 [5].

3.- Data analysis tools

Other R libraries used for the data analyses were: speedyseq v. 0.5.3.9018 (github.com/mikemc/speedyseq), magrittr v. 2.0.2 [6], reshape2 v. 1.4.4 [7], microbiome v.1.16.0 [8] and janitor v. 2.1.0 [9]. Plots were created with ggplot2 v. 3.3.5 [10], ggridges v. 0.5.3 [11], multipanelfigure v. 2.1.2 [12], ggforce v. 0.3.3 [13], ggpubr v. 0.4.0 [14], ggpmisc v.0.4.7 [15], scales v. 1.2.0 [16], ggpubr v. 0.4.0 and multcompView v. 0.1-8 [17].

Methodological limitations of the approach chosen

We identified different bias that could affect our estimates of prokaryotic growth rate. First, there is a taxonomic bias [18], i.e. differences in the efficiency of amplification of different taxa, but this is expected to be constant over time, and as such it should not affect the slope of the linear regression (i.e. growth) of 'pseudoabundance' over time [18]. Similarly, the variation in 16S rRNA gene copy number among different taxa [33, 57, 58] could affect the estimated growth rates, but as this is likely to be constant over time for each ASV, then the calculation of single-ASVs growth rates (i.e. the slope of the growth curve) should be barely affected by this bias. This was confirmed

with the significant correlation observed between the single-ASV-based growth rates normalized by 16S rRNA gene copy number versus those that were not normalized (Fig. S4). This bias would only affect our results in the case of ASVs growing so fast that could have more than one copy of their genome at the same time, which would lead to an overestimation of their relative abundance in some samples. Yet, experimental replication would minimize this potential bias. Furthermore, comparison of our growth rates values with other PCR-free estimates based on total cell-counts and metagenome OTUs yielded significant correlations and consistent trends (Fig. 1F), suggesting that the effect of PCR bias [22] was minimal. Likewise, the growth rate values of different bacterial populations based on CARD-FISH probes correlated in most cases with the single-ASV based growth rates (Fig. S5, Table S5). For some groups, the correlation was not significant, which may be explained by the fact that some CARD-FISH probes lack specificity or may not label all ASVs present in our experiments [23], and that in some cases they also target non-growing cells [24]. Whereas metagenomic approximations like the peak-to-through [25] or codon usage bias [26, 27] would be another option for assessing growth, they have their own limitations for marine prokaryotes [28]. Moreover, the codon usage bias method calculates maximal growth rates, whereas our intention was to assess the range of experimentally determined growth rates. In addition, these metagenome-based methods only work for the most abundant prokaryotes, neglecting an immense fraction of prokaryotic diversity. The consistency among the different tested methods provides compelling evidence that our results are solid.

Supplementary results

Detailed observations for different ASVs of the controlling factors affecting their growth along the year. For example, ASV10 (*Alteromonadaceae*) was highly affected by nutrient availability in spring and fall but not in winter and summer. However, its closely related taxon, ASV32 (*Alteromonadaceae*), presented a different trend (Fig. 4), and no consistency was observed at the family or order level (Fig. S9 and Fig. S10, respectively). Yet, some noticeable seasonal patterns could be observed: the growth rates of the Alteromonadales were limited by the availability of light in winter (Fig. S10), and growth of the Vibrionales had different limiting factors in the different seasons, controlled by the availability of light (in the control treatment), by viruses' pressure in winter, and by nutrient limitation in summer (Fig. S10). For some ASVs belonging to

Flavobacteriaceae (Fig. 4), the growth was more controlled by grazers in winter and spring, and this was clear at the family and order levels (Fig. S9 and S10). However, in summer, the growth of *Flavobacteriaceae* and Flavobacteriales was more determined by the availability of resources. At the family level other similar patterns could be observed (Fig. S9), with changing growth limiting factors over the different seasons for each taxonomic family. In contrast, we identified some taxonomic coherence for ASVs belonging to the *Rhodobacteraceae* family: ASV4 was highly positively impacted by the removal of the top-down control almost all the year, similarly to its closely related ASV9 (Fig. 4), these observations were also apparent at high taxonomic ranks in *Rhodobacteraceae* (Fig. S9) and Rhodobacterales (Fig. S10). Finally, *Sphingomonadaceae* (Fig. S9) or Sphingomonadales (Fig. S10) growth rates were the ones more negatively affected by viruses throughout most of the year.

References

1. Sánchez O, Ferrera I, Mabrito I, Gazulla CR, Sebastián M, Auladell A, et al. Seasonal impact of grazing, viral mortality, resource availability and light on the group-specific growth rates of coastal Mediterranean bacterioplankton. *Sci Rep* 2020; **10**: 19773.
2. Martin M. Cutadapt removes adapter sequences from high-throughput sequencing reads. *EMBnet.journal* 2011; **17**: 10.
3. Callahan BJ, McMurdie PJ, Rosen MJ, Han AW, Johnson AJA, Holmes SP. DADA2: High-resolution sample inference from Illumina amplicon data. *Nat Methods* 2016; **13**: 581–583.
4. Wright ES. Using DECIPHER v2.0 to analyze big biological sequence data in R. *R J* 2016; **8**: 352–359.
5. Rognes T, Flouri T, Nichols B, Quince C, Mahé F. VSEARCH: A versatile open source tool for metagenomics. *PeerJ* 2016; **2016**: 1–22.
6. Bache SM, Wickham H. magrittr: a forward-pipe operator for R. *R Packag version* 2014; **1** https://cran.r-project.org/package=magrittr.
7. Wickham H. Reshaping Data with the {reshape} Package. *J Stat Softw* 2007; **21**: 1–20.
8. Lahti L, Shetty S. microbiome R package http://microbiome.github.com/microbiome.
9. Firke S. janitor: Simple Tools for Examining and Cleaning Dirty Data. *R Packag version* 2021. https://cran.r-project.org/package=janitor
10. Wickham H, Chang W, Wickham MH. ggplot2: Elegant Graphics for Data Analysis. *Springer-Verlag New York* 2016; **2**: 1–189.
11. Aldahmani S, Zoubeidi T, Aldahmani MS. Package 'GGRidge'. *R Packag version* 2021. https://CRAN.R-project.org/package=ggridges
12. Graumann J, Cotton R. Multipanelfigure: Simple assembly of multiple plots and images into a compound figure. *J Stat Softw* 2018; **84**.
13. Pedersen TL. ggforce: Accelerating 'ggplot2'. *R Packag version* 2021. https://cran.r-project.org/package=ggforce
14. Kassambara A. ggpubr: 'ggplot2' Based Publication Ready Plots. *R Packag version* 2020. https://cran.r-project.org/package=ggpubr
15. Aphalo PJ. ggpmisc: Miscellaneous Extensions to 'ggplot2'. *R Packag version* 2022. https://cran.r-project.org/package=ggpmisc

16. Wickham H, Seidel D. scales: Scale Functions for Visualization. *R Packag version* 2022. https://cran.r-project.org/package=scales

17. Graves S, Piepho H-P, with help from Sundar Dorai-Raj LS. multcompView: Visualizations of Paired Comparisons. *R Packag version* 2019. https://cran.r-project.org/package=multcompView

18. McLaren MR, Nearing JT, Willis AD, Lloyd KG, Callahan BJ. Implications of taxonomic bias for microbial differential-abundance analysis. *bioRxiv* 2022; 2022.08.19.504330.

19. Stoddard SF, Smith BJ, Hein R, Roller BRK, Schmidt TM. rrnDB: Improved tools for interpreting rRNA gene abundance in bacteria and archaea and a new foundation for future development. *Nucleic Acids Res* 2015; **43**: D593–D598.

20. Gao Y, Wu M. Accounting for 16S rRNA copy number prediction uncertainty and its implications in microbial diversity analyses. *bioRxiv* 2023; 2021.08.31.458422.

21. Soppa J. Polyploidy and community structure. *Nat Microbiol* 2017; **2**: 1–2.

22. Pinto AJ, Raskin L. PCR biases distort bacterial and archaeal community structure in pyrosequencing datasets. *PLoS One* 2012; **7**.

23. Acinas SG, Ferrera I, Sarmento H, Díez-Vives C, Forn I, Ruiz-González C, et al. Validation of a new catalysed reporter deposition- fluorescence in situ hybridization probe for the accurate quantification of marine Bacteroidetes populations. *Environ Microbiol* 2015; **17**: 3557–3569.

24. Alonso-Sáez L, Sánchez O, Gasol JM. Bacterial uptake of low molecular weight organics in the subtropical Atlantic: Are major phylogenetic groups functionally different? *Limnol Oceanogr* 2012; **57**: 798–808.

25. Brown CT, Olm MR, Thomas BC, Banfield JF. Measurement of bacterial replication rates in microbial communities. *Nat Biotechnol* 2016; **34**: 1256–1263.

26. Vieira-Silva S, Rocha EPC. The systemic imprint of growth and its uses in ecological (meta)genomics. *PLoS Genet* 2010; **6**: e1000808.

27. Weissman JL, Hou S, Fuhrman JA. Estimating maximal microbial growth rates from cultures, metagenomes, and single cells via codon usage patterns. *Proc Natl Acad Sci U S A* 2021; **118**: 1–10.

28. Long AM, Hou S, Ignacio-Espinoza JC, Fuhrman JA. Benchmarking microbial growth rate

predictions from metagenomes. *ISME J* 2020; 183–195.

29. Daimsl H, Bruhll A, Amann R, Schleifer K, Wagner M. APPlIED MICROBIOlOGY The Domain-specific Probe EUB338 is Insufficient for the Detection of all Bacteria : Development and Evaluation of a more Comprehensive Probe Set. 1999; **444**: 434–444.

30. Amann RI, Binder BJ, Olson RJ, Chisholm SW, Devereux R, Stahl DA. Combination of 16S rRNA-targeted oligonucleotide probes with flow cytometry for analyzing mixed microbial populations. *Appl Environ Microbiol* 1990; **56**: 1919–1925.

Supplementary tables

Tables are shown in an additional excel file.

Table legends

Table S1: Physicochemical and biological parameters at the beginning (t_0) and the end of the experiments (t_4).

Table S2: Number of negative rates for the 5 taxonomic Orders that had the highest number of significant ($p < 0.05$) negative rates.

Table S3: Single ASV-based growth rates (d^{-1}) calculated in these experiments for all treatments at the different seasons and the taxonomy of each ASV.

Table S4: Taxonomy and maximal growth rate of the top 20 single-ASV-based growth rates values calculated in these experiments.

Table S5: Summary of correlations between single ASV-based growth rates and growth rates estimated with CARD-FISH probes targeting different groups (see methods for details). The correlations were performed with mean values for each condition (season and treatment) for all ASVs of the given phylogenetic group versus CARD-FISH-estimated abundance-based growth rates for each condition. Before performing the correlations, normality was checked with the Shapiro-test. In case of normality, we used a parametric test (Pearson), and in case of no normality we used a non-parametric test (Spearman).

Table S6: Growth rates per treatment and season at the ASV level of those organisms presented in figure 4 which are those that had more than 10 growth rates to compare between treatments and seasons.

Table S7: Mean (and standard deviation) growth rates per treatment and season at the family level as plotted in figure S9.

Table S8: Mean (and standard deviation) growth rates per treatment and season at the order level as plotted in figure S10.

Table S9: Summary of the growth rates range at the taxonomic level of genus for the shared genera between the Fecskeová et *al.*, (2021) study and the present study, specifically the spring experiment for treatments control and predator reduced both with natural day light regime.

Supplementary figures

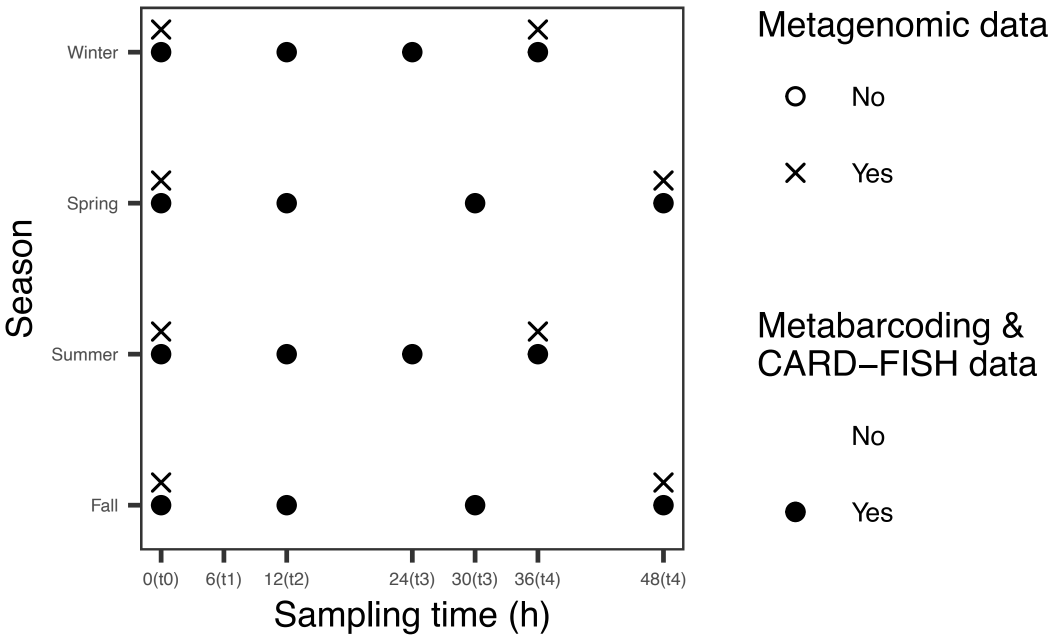

Figure S1: Scheme of the samples used for growth rates calculation and the hours at which they were taken.

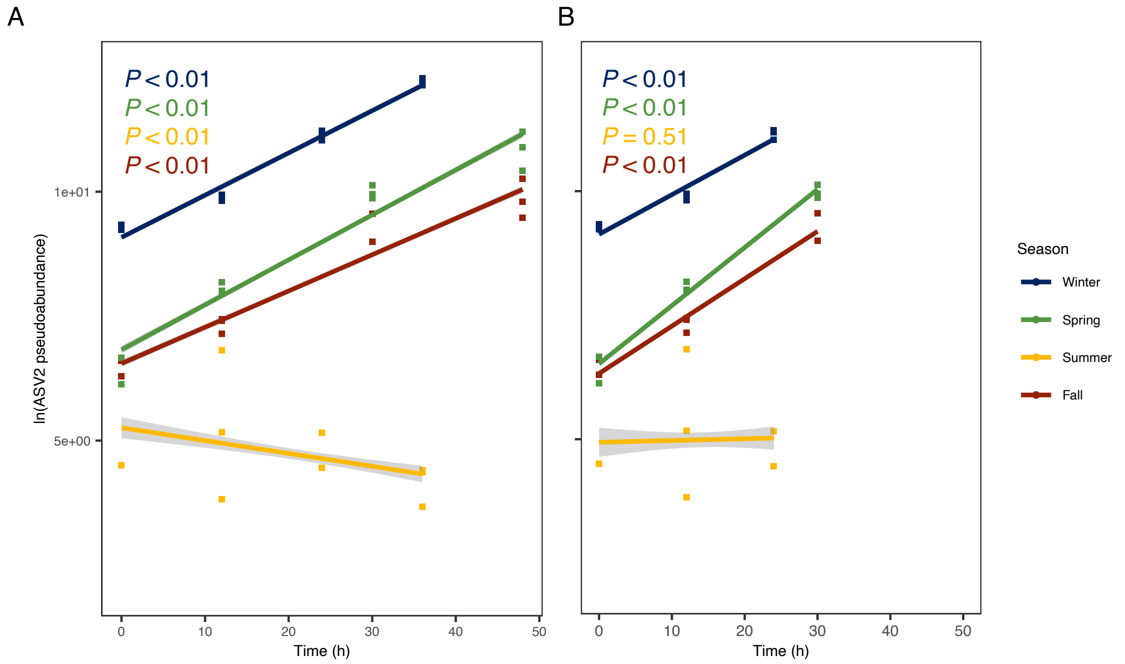

Figure S2. Example of calculation of growth rates, specifically for ASV2 in the DL treatment in the four seasons. The X axis represents time (h) and the Y axis represents pseudoabundance (relative abundance times the total prokaryotic abundance in the sample) as a natural logarithm. Assuming exponential growth, the slope of the linear regression represents the growth rate, in units of $time^{-1}$. The line shows a linear regression using the three replicates for each condition (season and treatment). Panel A shows the linear regression using data of the four times sampled, while in panel B using the first three ones. We systematically compared these two estimates and retained the one that was significant (p-value < 0.05) and highest.

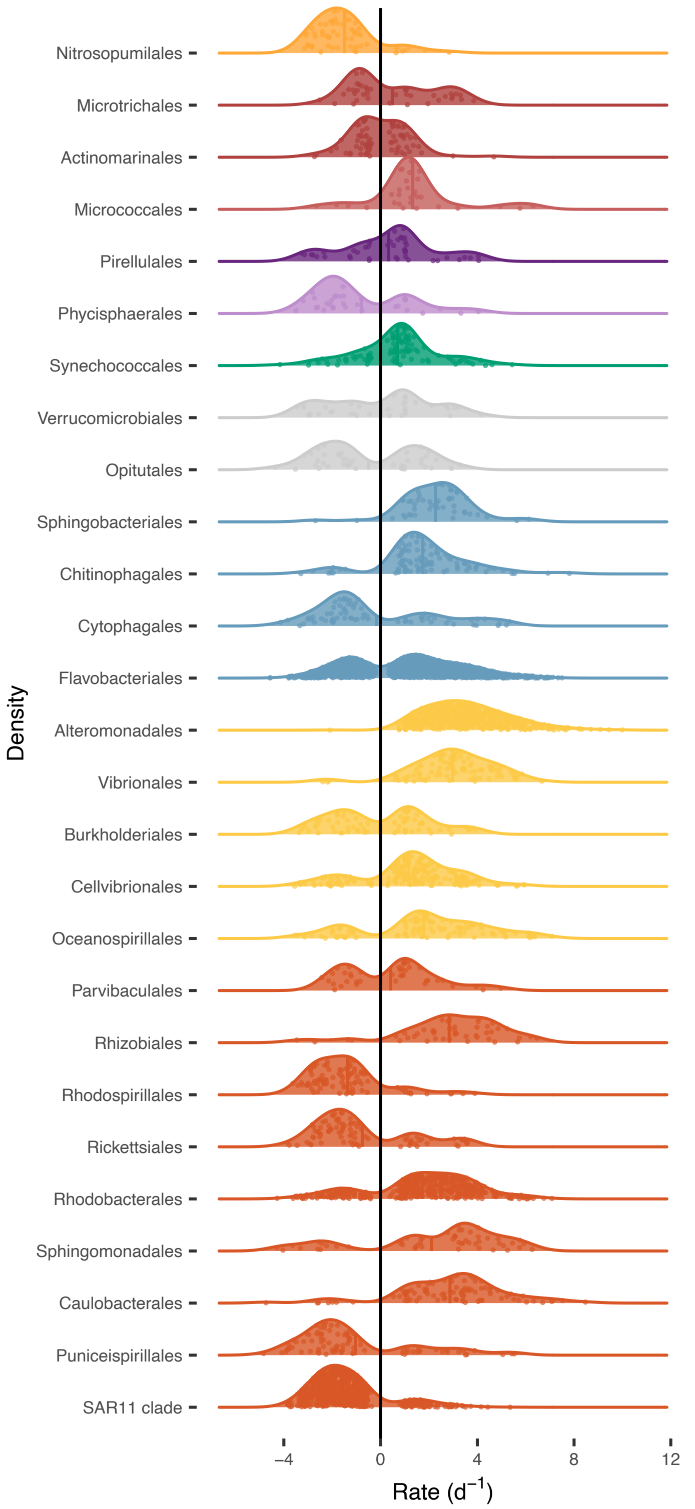

Figure S3. Density distribution of positive and negative rates for those Orders that presented at least 25 significant ($p < 0.05$) rates over all the experiments. The color indicates all Orders that belong to the same taxonomic Class. The black vertical line indicates the 0 rate. In the X axis we present the rate (d^{-1}) and in the Y axis the density.

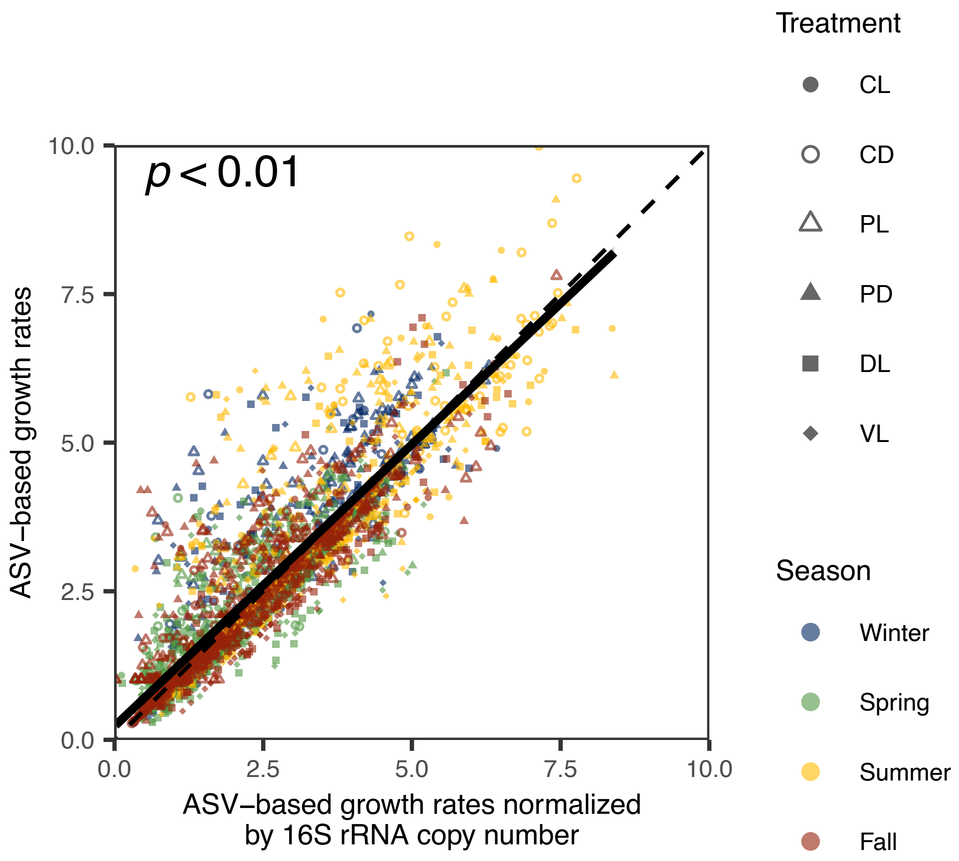

Figure S4. Correlation between single ASV-based growth rates with reads previously normalized by 16S rRNA gene copy number versus the ones from reads without this normalization. We tested the correlation using Spearman, as normality was not confirmed by the Shapiro test. Dashed line indicates the 1:1 relationship, and the p-value indicates the significance of the correlation. Each point represents an ASV growth rate and its color indicates the season while the shape indicates the treatment they belong to.

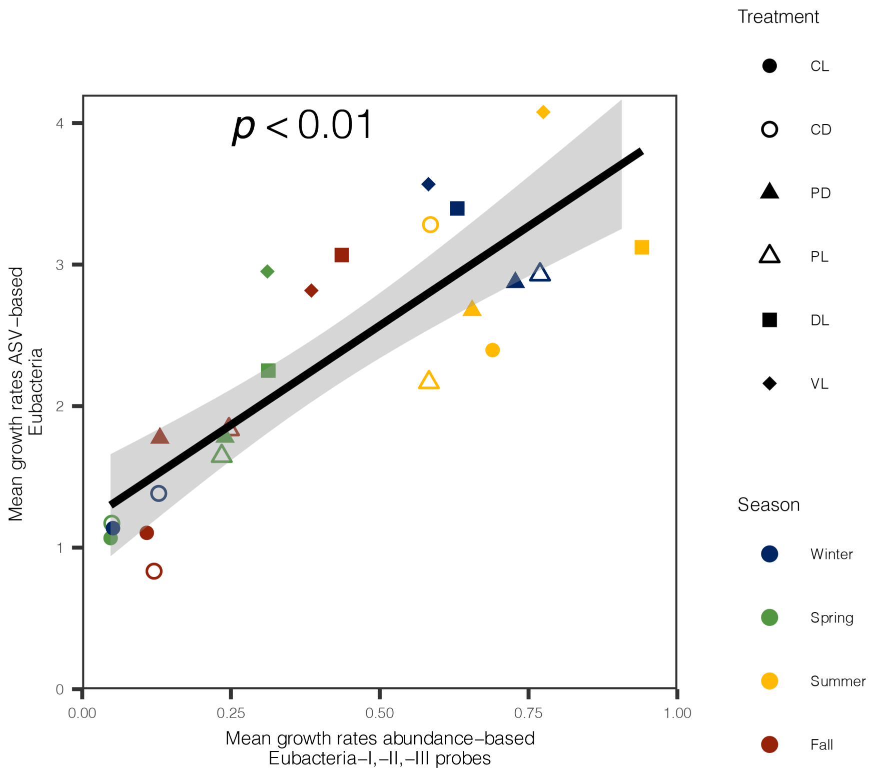

Figure S5. Correlation between single ASV-based growth rates and growth rates obtained by using CARD-FISH probes Eubacteria I, II and III [29, 30] using the mean growth rate values per condition (season and treatment).

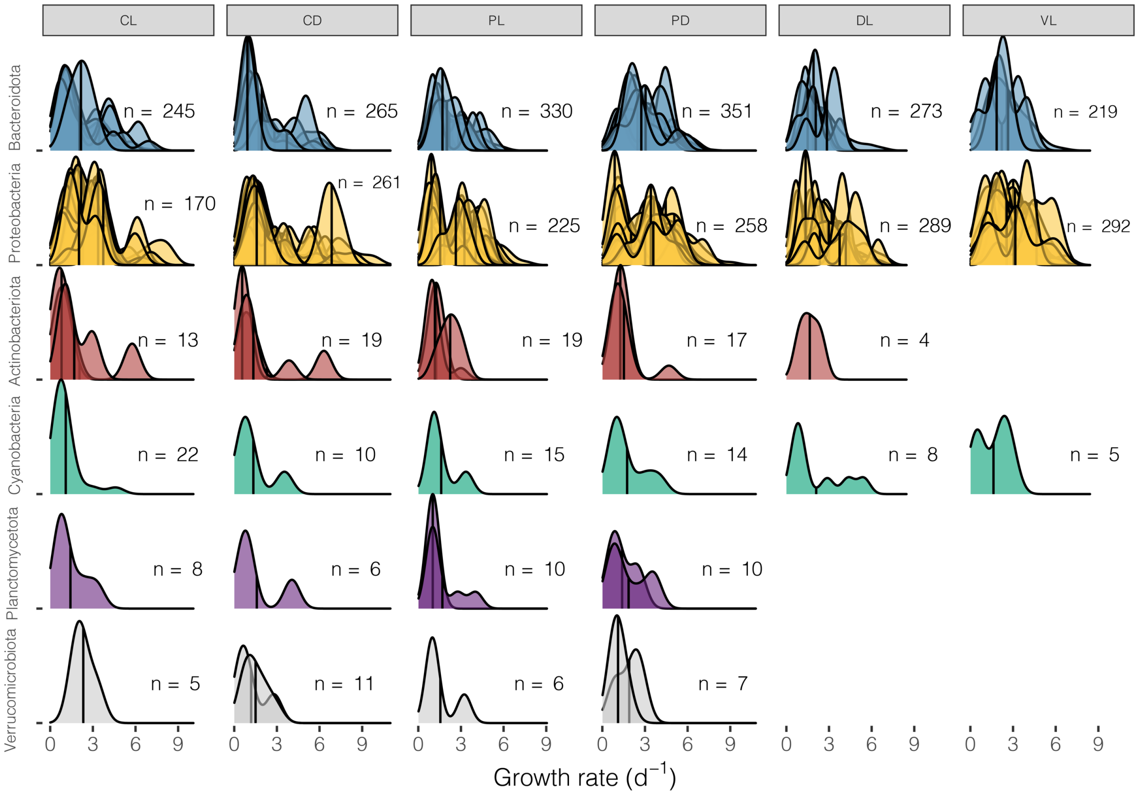

Figure S6. Growth rate distribution patterns grouped by families belonging to each phylum over the year separated by treatments plotted in a ridgeline plot, which presents density estimates. We only plotted those families that had at least 10 growth rate values in all the experiments. This filter was used to simplify the visualization of the plot.

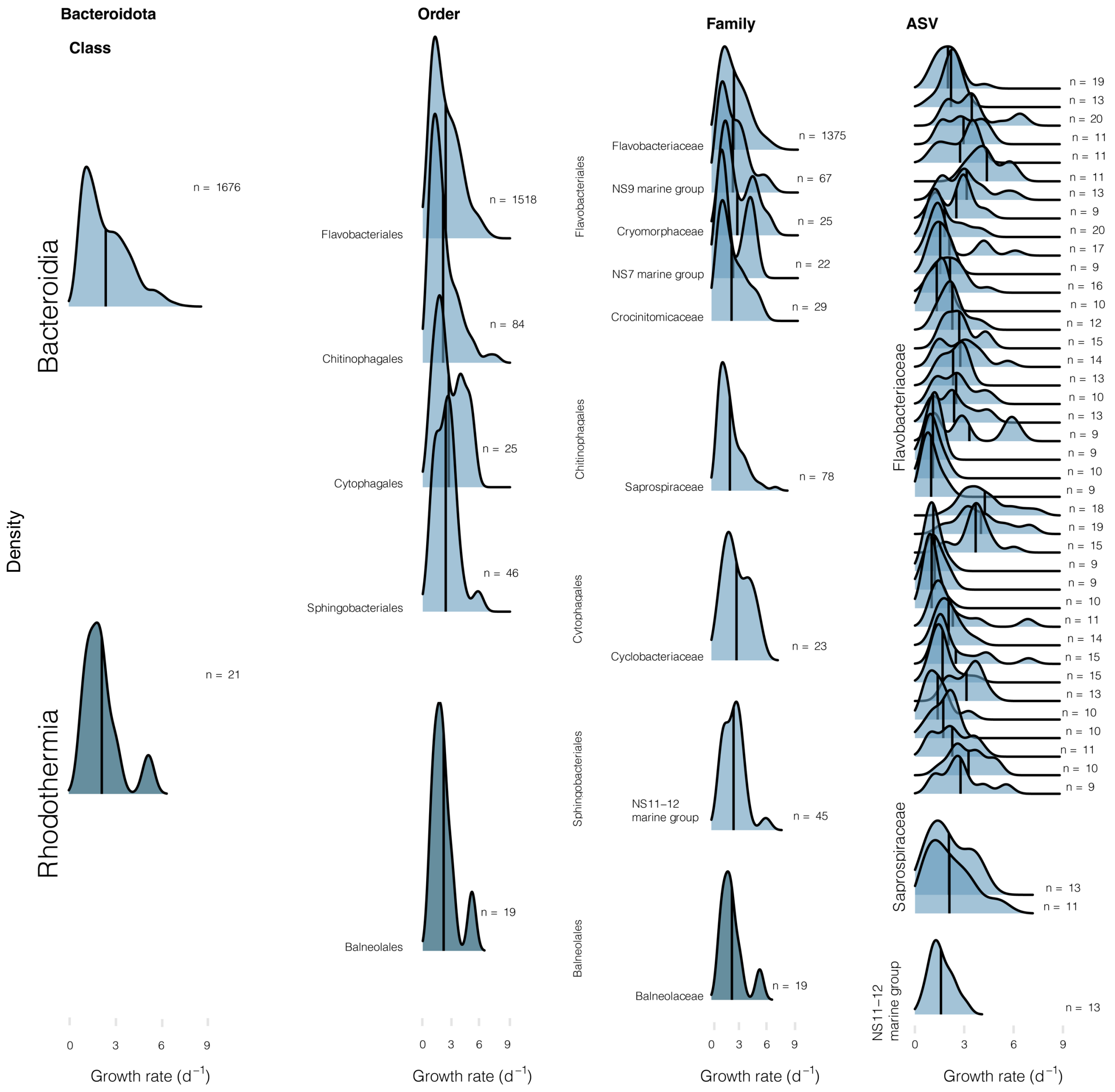

Figure S7. Growth rate distribution patterns of phylum Bacteroidota plotted in a ridgeline plot, which presents density estimates. Here, each column represents a different taxonomic level; from left to right: class, order, family and ASV, and the X axis shows the distribution of growth rates (d^{-1}). The dataset was filtered with the following parameters: classes, orders and families with more than 10 growth rate values and ASVs with more than 8 growth rates. This filter was used to simplify the visualization of the plot.

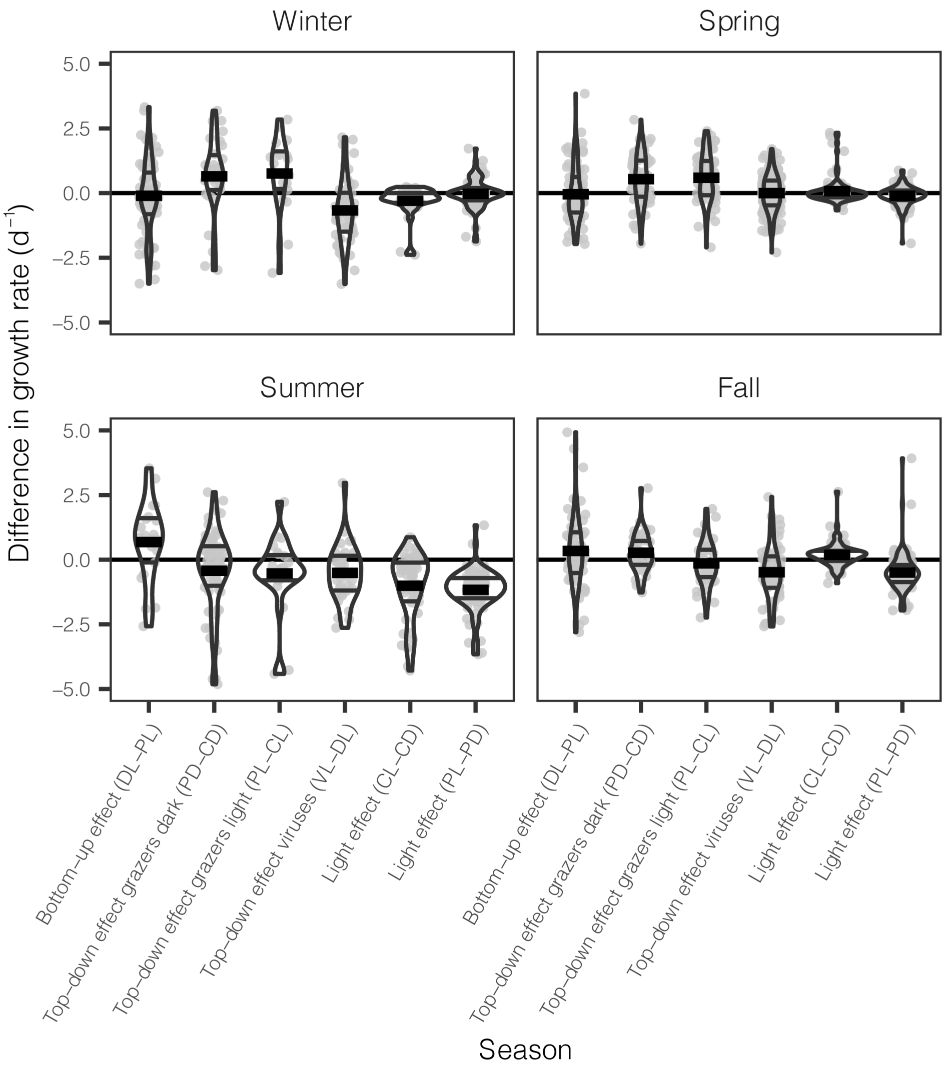

Figure S8. Summary of the effects of the controlling factors on growth rate at the ASV level for all limiting factors over the year. Points represent the differences between the growth rate values of each ASV from different treatments at each season. The wide black lines indicate mean values for all ASVs growth rate differences, and the thin black lines indicate quartiles.

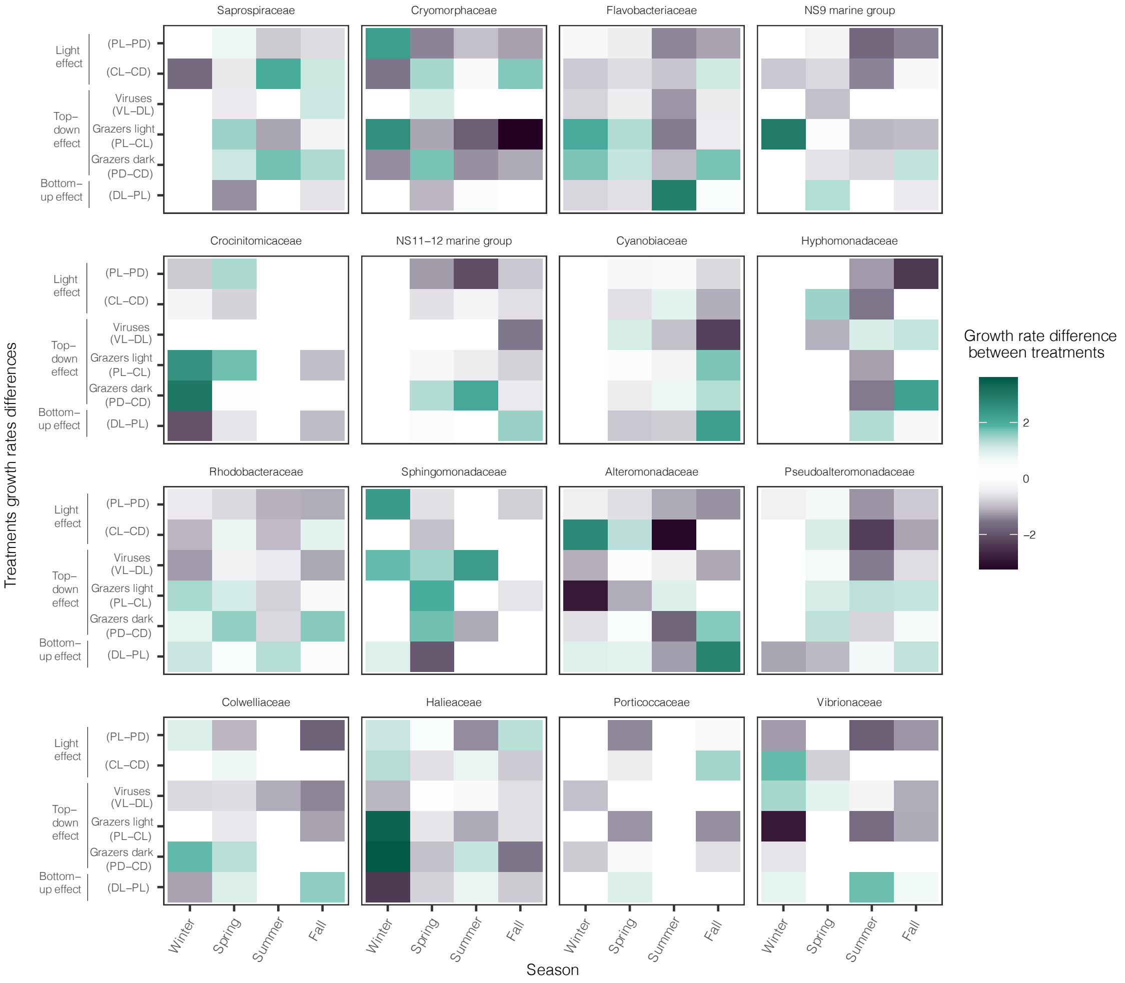

Figure S9. Summary of the effect of the various limiting factors on growth rates of different families across seasons (only those families with at least 10 values over all the treatments and seasons are shown). On the X axis the seasons are represented, and on the Y axis the different effects, presented as the difference between the mean growth rate from one treatment to another. Color coding indicates the following. Green means a positive effect, and grey means a negative effect of that type of limiting factor for that phylogenetic family. The summary of mean and standard deviation is presented in the supplementary information (Table S7).

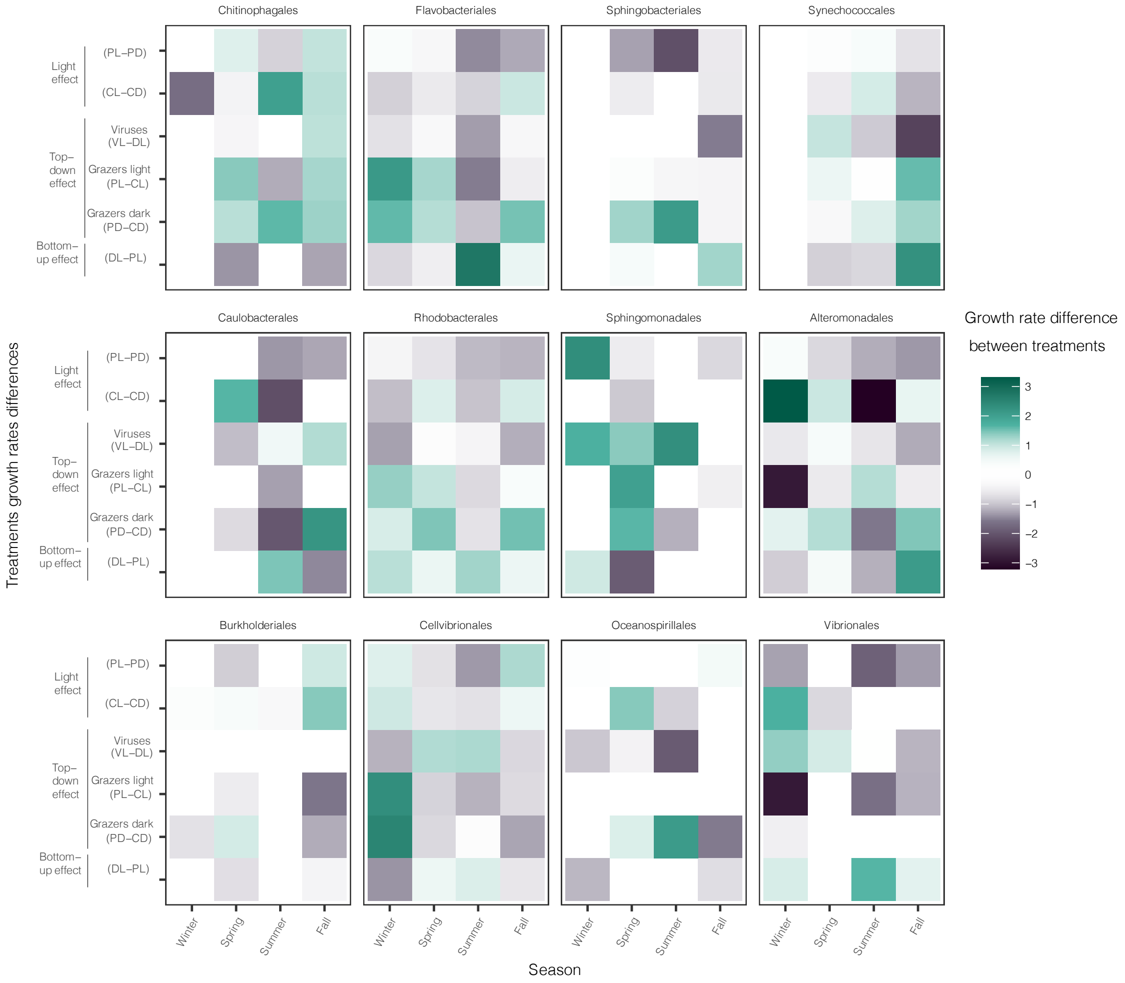

Figure S10. Summary of treatment effects on growth rates values at different Orders that had at least 12 values over all the treatments and seasons. On the X axis seasons are represented, and on the Y axis the difference between the mean growth rates from different treatments is represented by color. Green means positive effect, and grey means negative effect of that type of limiting factor for that phylogenetic order. The summary of mean and standard deviation is presented in supplementary information (Table S8).

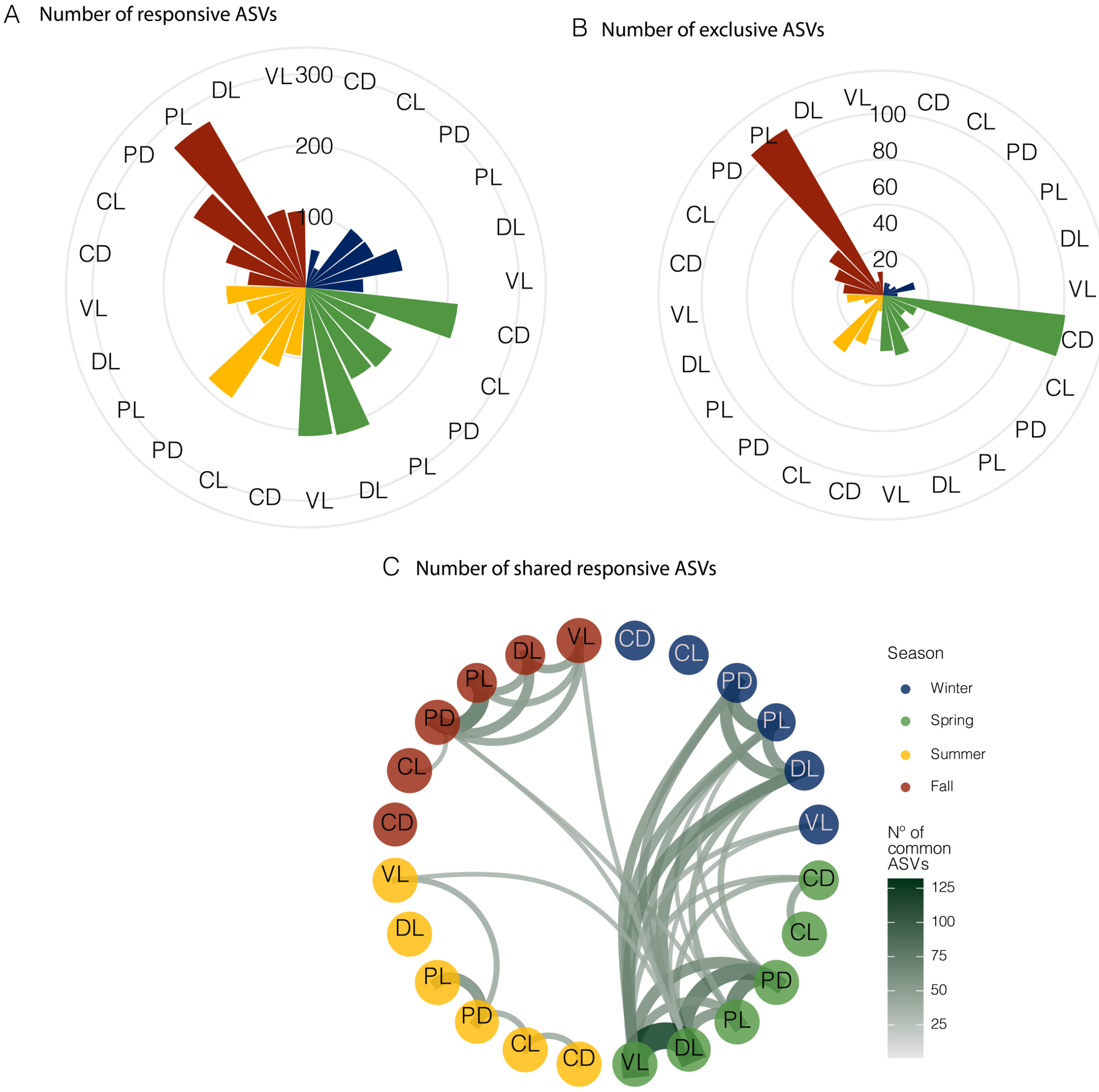

Figure S11. Analysis of responsive ASVs in the different treatments and seasons. We considered that an ASV was responding to a treatment if it presented a growth rate >1 d^{-1}. A) Absolute number of responsive ASV in each treatment. B) Number of exclusive (i.e. ASVs that only responded to one treatment and season) ASVs growing per each sample and treatment. C) Absolute number of responsive ASVs shared between treatments and seasons. Line width and color represent the number of shared ASV between the linked samples. Only connections higher than 30% of the maximum number of shared ASVs (39 ASVs) are plotted. Colors denote the different seasons.

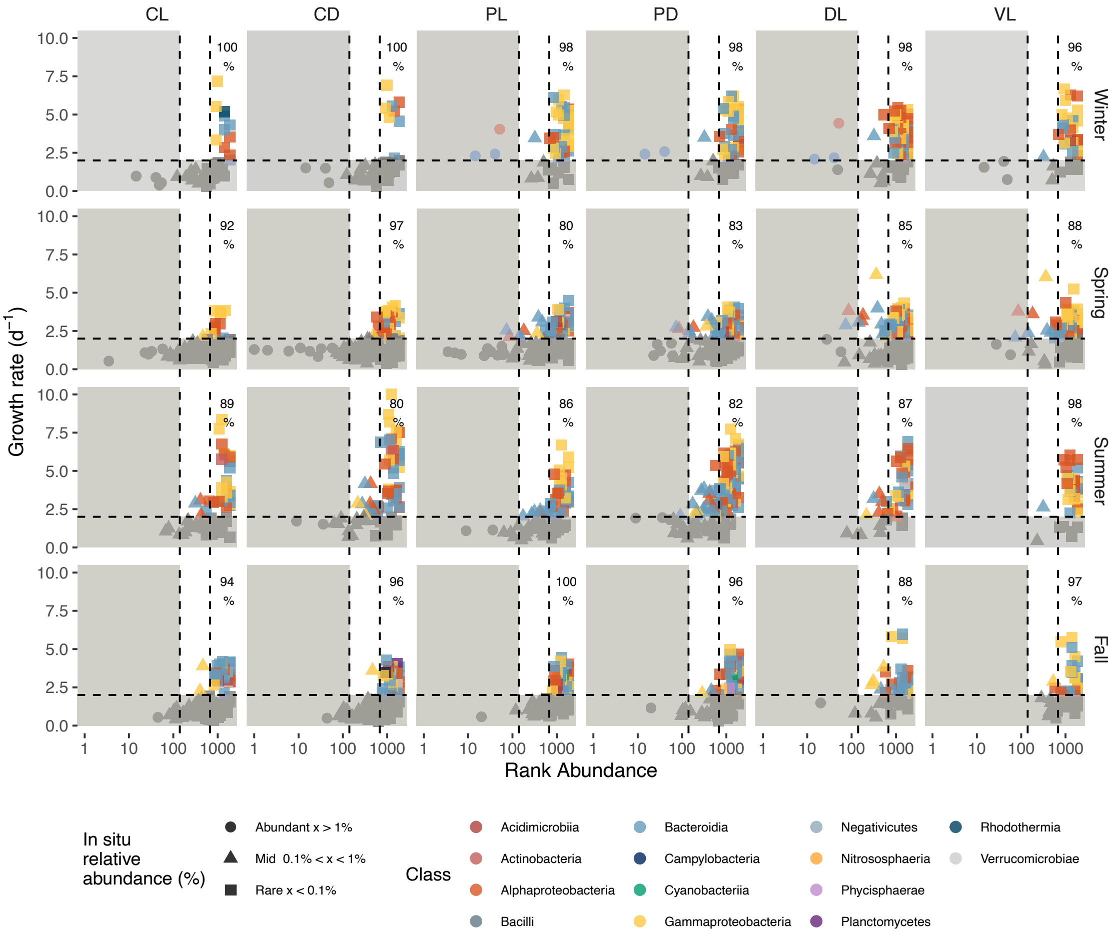

Figure S12. Relationship between relative abundance in the natural community and growth rates in the different treatments. The X axis is the rank abundance of ASVs ordered by their abundance in the *in situ* communities. Note that the X-Axis has been log-transformed for visualization. In the Y axis we present the growth rate (d^{-1}). Dashed vertical lines indicate relative abundances of 1% and 0.1% of the natural community, and the horizontal dashed line a growth rate of 2 (d^{-1}). The number indicates the percentage of most-responsive taxa that were initially very rare. Dot color indicate taxonomic class.

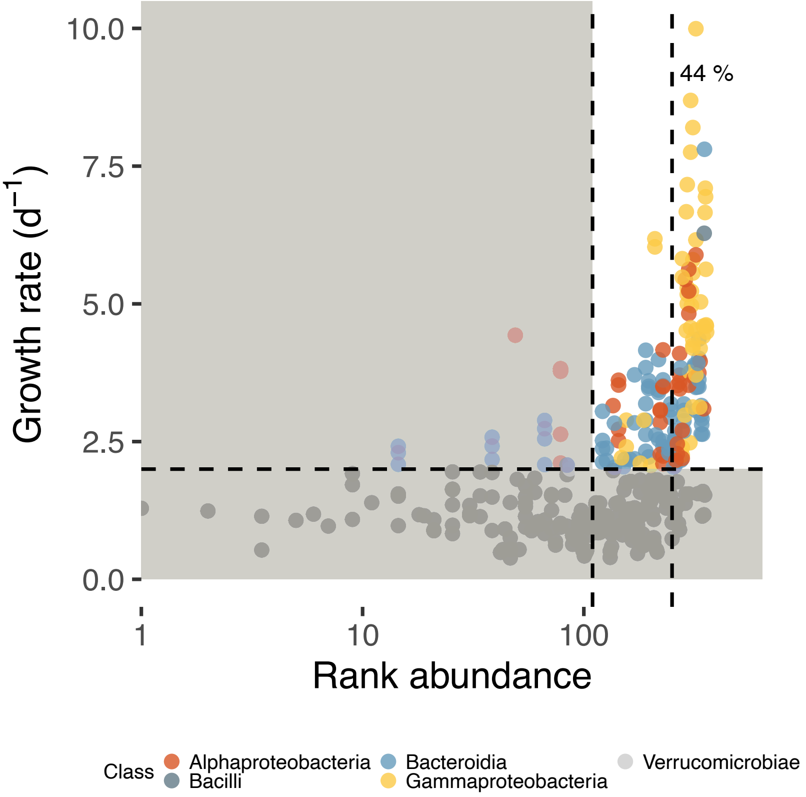

Figure S13. Successful rare-most-responsive ASVs defined as those ASVs that represented less than 1% of the community relative abundance at the start of the experiments but reached values > 1% at the end of the experiments, with a growth rate value higher than 2 d^{-1}. The X axis represents the rank abundance of responsive ASVs, ordered by their abundance in *the in situ* communities (where 1 represents the most abundant ASV). Note that the X-Axis has been log-transformed for visualization. In the Y axis we present the growth rate (d^{-1}). The dashed vertical lines indicate the 1% and 0.01% of the relative abundance at the natural community, respectively. The 44% value indicates the percentage of successful rare-most-responsive ASVs (ASVs that had a growth rate > 2 d^{-1} and less than 0.1% of the community at the beginning of the experiments and ended up being more than 1% of the community at the end of the experiments in at least one experiment and season) from the total rare-most-responsive ASVs.

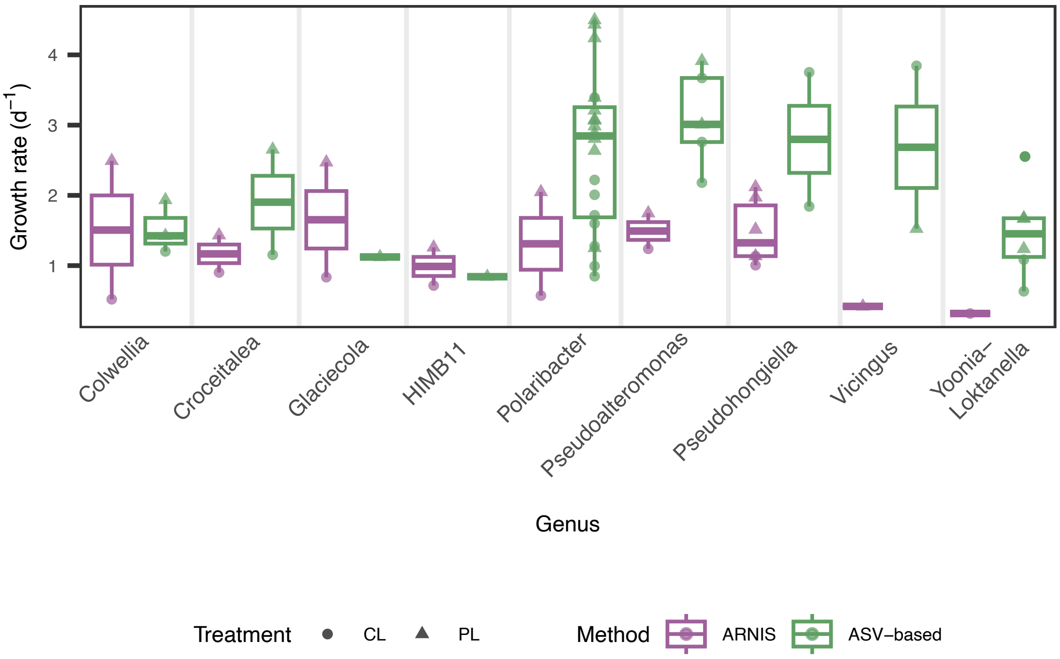

Figure S14. Boxplot illustrating the growth rates of shared genera between the Fecskeová et *al.*, (2021) study and the present study, specifically the spring experiment for treatments control and predator reduced, both with natural day light regime. Growth rates obtained via the ARNIS method pertain to the Fecskesová et *al.*, (2021) experiment conducted using water from the Middle Adriatic Sea, while ASV-based growth rates correspond to experiments conducted with water from the NW Mediterranean Sea. Treatment conditions are denoted by point shape, while the method used for growth rate calculation is represented by color.
